# Biocompatible Anionic Polymeric Microspheres as Priming Delivery System for Effetive HIV/AIDS Tat-Based Vaccines

**DOI:** 10.1371/journal.pone.0111360

**Published:** 2014-10-30

**Authors:** Fausto Titti, Maria T. Maggiorella, Flavia Ferrantelli, Leonardo Sernicola, Stefania Bellino, Barbara Collacchi, Emanuele Fanales Belasio, Sonia Moretti, Maria Rosaria Pavone Cossut, Roberto Belli, Erika Olivieri, Stefania Farcomeni, Daniela Compagnoni, Zuleika Michelini, Michela Sabbatucci, Katia Sparnacci, Luisa Tondelli, Michele Laus, Aurelio Cafaro, Antonella Caputo, Barbara Ensoli

**Affiliations:** 1 National AIDS Center, Istituto Superiore di Sanità, Rome, Italy; 2 Department of Environmental and Live Science, University of East Piemonte, Alessandria, Italy; 3 I.S.O.F., Consiglio Nazionale delle Ricerche, Bologna, Italy; 4 Department of Molecular Medicine, University of Padova, Padova, Italy; University of Alabama, United States of America

## Abstract

Here we describe a prime-boost regimen of vaccination in *Macaca fascicularis* that combines priming with novel anionic microspheres designed to deliver the biologically active HIV-1 Tat protein and boosting with Tat in Alum. This regimen of immunization modulated the IgG subclass profile and elicited a balanced Th1-Th2 type of humoral and cellular responses. Remarkably, following intravenous challenge with SHIV89.6P_cy243_, vaccinees significantly blunted acute viremia, as compared to control monkeys, and this control was associated with significantly lower CD4^+^ T cell depletion rate during the acute phase of infection and higher ability to resume the CD4^+^ T cell counts in the post-acute and chronic phases of infection. The long lasting control of viremia was associated with the persistence of high titers anti-Tat antibodies whose profile clearly distinguished vaccinees in controllers and viremics. Controllers, as opposed to vaccinated and viremic cynos, exhibited significantly higher pre-challenge antibody responses to peptides spanning the glutamine-rich and the RGD-integrin-binding regions of Tat. Finally, among vaccinees, titers of anti-Tat IgG1, IgG3 and IgG4 subclasses had a significant association with control of viremia in the acute and post-acute phases of infection. Altogether these findings indicate that the Tat/H1D/Alum regimen of immunization holds promise for next generation vaccines with Tat protein or other proteins for which maintenance of the native conformation and activity are critical for optimal immunogenicity. Our results also provide novel information on the role of anti-Tat responses in the prevention of HIV pathogenesis and for the design of new vaccine candidates.

## Introduction

Several studies in nonhuman primates demonstrated that live attenuated viral/bacterial vectors possess very attractive features for the development of an effective anti-HIV/AIDS vaccine since they target specific antigen presenting cells, stimulate the innate immunity and induce/expand antiviral responses against the vaccine antigen(s) [1], [2], [3], [4]. Nevertheless, none of the live attenuated vectors tested so far has proven to be an ideal vehicle for HIV-1 vaccine antigens not only due to the generation of potent anti-vector immune responses but also because the vector itself can express factor(s) having a negative impact on the antigen-driven immunity, as demonstrated for the MVA vector [5], [6]. The increasing emphasis for improved vaccine safety has led to the development of subunit vaccine approaches based on highly purified proteins characterized by a well-defined composition and higher safety. However, very often these proteins are instable and difficult to handle without cold chain infrastructures. Consequently, in order to be immunogenic, they require the use of suitable chemical adjuvants and/or delivery systems of which only few have been licensed for human use. Furthermore, the development of adjuvant and/or delivery systems to improve both antibody and T-cell mediated cell responses still remains a challenge.

Non-reactogenic polymeric microspheres are thought to be an important tool to deliver vaccine antigens, since they are devoid of the toxic effects shown by other adjuvants and do not elicit anti-vector responses as observed with biological vectors. Incorporation of proteins in poly(dl-lactide) (PLA) and poly(dl-lactide-co-glycolide) (PLGA) biodegradable microspheres have been shown to elicit strong, long-term, immune responses in preclinical models [7], [8], [9], [10], [11]. However, although proteins encapsulated into PLA or PLGA matrices may be protected from unfavourable conditions (e.g. pH, bile salts and proteolytic enzymes), a common problem with these delivery approaches is the instability or the degradation of the entrapped antigen [12], [13], [14]. Conversely, surface adsorption strategies on biocompatible or biodegradable particles have been shown to have higher loading ability and preservation of antigen bioactivity as compared to encapsulation approaches [15], [16], [17], [18], [19], [20], [21]. In this scenario, we have previously described novel biocompatible microparticles (named H1Ds) having a core-shell structure with an inner core constituted by poly(methylmethacrylate) (PMMA) and a highly hydrophilic outer shell composed of a hydrosoluble co-polymer, namely poly(methacrylic acid-*st*-ethyl acrylate) copolymer (Eudragit L100-55), tightly linked to the core and bearing carboxylic groups [22]. The synthetic procedure for the generation of H1Ds allows a single step, low cost production of highly reproducible, homogenous in size and size distribution, surfactant-free particles able to be loaded with up to 9% (w/w) of basic proteins. H1Ds can load protein(s) and have the ability to enter splenocytes, monocytes, monocyte-derived dendritic and epithelial cells with high efficiency [23]. Further, adsorption of the biologically active HIV-1 Tat protein on the surface of H1Ds prevents heat- and light-induced Tat oxidation and loss of biological activity, thus increasing the stability and shelf-life of the Tat protein [24]. Thus, because of their features, H1Ds represent a very attractive system to deliver vaccine antigens, particularly when maintenance of the native conformation is essential for immunogenicity. In fact, we have reported that immunization with a new vaccine formulation composed by these microspheres and the HIV-1 Tat protein was safe and induced robust and long-lasting cellular and humoral responses in mice after systemic and/or mucosal administration [25].

Studies in preclinical and in clinical trials have indicated that the HIV-1 Tat protein is a safe and reliable candidate for preventative and/or therapeutic immunization against HIV/AIDS [26], [27], [28], [29], [30], [31] (http://www.hiv1tat-vaccines.info). Hence, the safety, immunogenicity and efficacy of a vaccine regimen based on priming with the Tat protein adsorbed onto H1D anionic microspheres (Tat/H1Ds) followed by boost with Tat in Alum was investigated in the *Macaca fascicularis* model of HIV/AIDS infection. We report that Tat/H1D prime-Tat/Alum boost regimen generated humoral (IgM, IgG) and both Th-1 and Th-2 type cellular responses. Of note, immunization significantly contained viral infection upon intravenous challenge with pathogenic cynos-derived SHIV89.6P_cy243_, and efficiently controlled CD4^+^ T cell decline resulting in a clear clinical benefit. Vaccinees exhibiting better control of viremia during the chronic phase of infection were those that had mounted anti-Tat antibody responses to relevant Tat domains upon vaccination. In particular, prechallenge anti-Tat IgG1 titers in vaccinees and, to a lesser extent IgG3 and IgG4 responses, were significantly associated with control of viral replication in the post-acute phase of the infection. Because of the limited number of monkeys in each group and the variability of the host’s genetic background, we were unable to determine the impact of MHC haplotypes on the level of protection observed.

## Materials and Methods

### Anionic microspheres

Core-shell microspheres, with a core constituted by poly(methylmethacrylate) (PMMA) and a highly hydrophilic shell composed of poly(methacrylic acid-*st*-ethyl acrylate) copolymer (Eudragit L100/55) were generated by dispersion polymerization, as described previously [18], [19], [20], lyophilized and stored at room temperature until use. The microspheres bear carboxylic groups able to reversibly bind biologically active basic proteins, such as the HIV-1 Tat protein, on their surface and are characterized by a very homogeneous size and size distribution. The microspheres (sample H1D) used in this study are characterized by a SEM diameter of 1.99 µm (±0.17), surface charge density of 57.4 COOH µmol/g and 9% (wt/wt) antigen loading ability. The endotoxin content of the particles was tested by the *Limulus* Amoebocyte Lysate (Pyrochrome, Cape Cod, Falmouth, MA, USA) and it was below the assay detection limit (<0.05 EU/µg).

### Tat protein

The monomeric biologically active Tat protein [86 aminoacids (aa)] of HIV-1 (HTLVIII-BH10) was produced in *Escherichia coli* (Advanced Bioscience Laboratories, Inc., Kensington, MD, USA) and tested for biological activity as described [32], [33]. Tat is photo-, air- and thermo-sensitive and oxidizes easily (due to the presence of seven cysteines in its sequence) when exposed to air, light and room temperature. Thus, to prevent oxidation, which causes aggregation of the bioactive monomers and loss of biological activity, the Tat protein was stored lyophilized at –80°C and resuspended at a relatively high concentration (2 mg/ml) in degassed commercial phosphate buffered saline (PBS) in the dark and on ice, immediately before use [25]. Endotoxin concentration of different lots of Tat was always below the detection limit (<0.05 EU/µg), as tested by the *Limulus* Amoebocyte Lysate analysis.

### Preparation of Tat/microspheres formulations

Tat (10 µg)/H1D (60 µg) complexes were prepared by mixing the Tat protein with the microspheres (resuspended in PBS at 2 mg/ml at least 24 hours earlier to allow complete hydration) under continuous stirring for 1 hour at 4°C. After incubation, complexes were collected at 14,000 rpm, resuspended in degassed sterile PBS (500 µl) and immediately inoculated intramuscularly (i.m.) in two sites for each monkey.

### Ethics statement

Adult male cynomolgus monkeys (*Macaca fascicularis*) (n = 18) of Mauritian origin (Primatology Research Center, Port Louis, Mauritius) were used in this study (CITES Permit no. MU1063, MU030442, MU00657, MU040563, MU02114). They tested negative for SIV, STLV-I, simian type-D retroviruses, Ebola & Marburg viruses, and simian Herpes B virus infections and housed in single cages in authorised P3 facility, at the National AIDS Center, Istituto Superiore di Sanità (ISS), in accordance with National (Ministry of Health DL n.116, 27/01/1992) and European guidelines for non-human primate care (EEC, Directive No. 86–609, Nov. 24, 1986 and Directive 2010/63 Sept. 22). The temperature was maintained at 21–23 °C and humidity ranged from 50 to 60% with 10–15 air changes per h. The light cycle was 12-h light/12-h dark. Animals were fed a commercial maintenance chow (Mucedola s.r.l., Settimo Milanese, Italy) supplemented biweekly with fresh fruit. Water was supplied *ad libitum*. Animal experiments were approved by the Quality and Safety Committee for animal trials of ISS and of Ministry of Health (D.M. 144/2004-C and D.M. 75/2006-B). All procedures were performed to minimize suffering, improve housing conditions, and to provide enrichment opportunities (e.g., varied food supplements, foraging and task-oriented feeding methods, interaction with caregivers and research staff, view of the other monkeys). Clinical procedures (immunizations, collection of blood, clinical examination, measurement of weight and rectal temperature) were performed on monkeys anesthetized with Zoletil-100 (10 mg/Kg). Peripheral blood samples for hematological, immunological and virological analysis were obtained in the morning before food administration. All monkeys were observed on a regular basis for behavior, major local reactions after immunizations, serum biochemical and hematological parameters, and clinical signs of disease. Hematological analyses were performed with an automatic particle counter (Coulter Onyx, Instrumentation Laboratory, Milan, Italy). The counts of white blood cells (i.e. neutrophil, eosynophil, basophil, lymphocytes and macrophages) were done using whole blood smears under light microscope examination after May Grünwald-Giemsa staining. PBMCs were purified from citrated whole blood on Ficoll-Hypaque (Pharmacia Bioteck AB, Uppsala, Sweden) gradients with a standard procedure and grown in RPMI1640 medium supplemented with 10% heat-inactivated FBS (Hyclone, Thermo Scientific Laboratories, Utah, USA). Plasma was separated from citrated blood by centrifugation at 850 g for 15 minutes, clarified at 2,000 g for 30 minutes at 4°C, aliquoted and stored at −80°C until use.

Monkeys enrolled in this protocol were matched for weight (vaccinees: mean gr. 4,672±408; controls: mean gr. 4,604±408) and CD4+ T cell counts (vaccinees: mean 1,441±400; controls: mean 1,661±491). Following the challenge infection, two controls (AC032 and AC739 at weeks 40 and 46 respectively) and one vaccinee (AF134, at week 46) were euthanized with humanitarian methods (Tanax 0.3 mg/Kg; Intervet International Gmbh, Germany) due the appearance of symptoms of simian AIDS (CD4+ T cell depletion, from 30–40% weight loss in three months, leukopenia, inverted leukocyte formula) and to avoid suffering. The vaccinated macaque AG269 was leukopenic and found death at week 26, likely for gastric dilatation, as observed at necroscopy.

### Monkeys, immunization schedule, virus challenge

**Figure 1:**
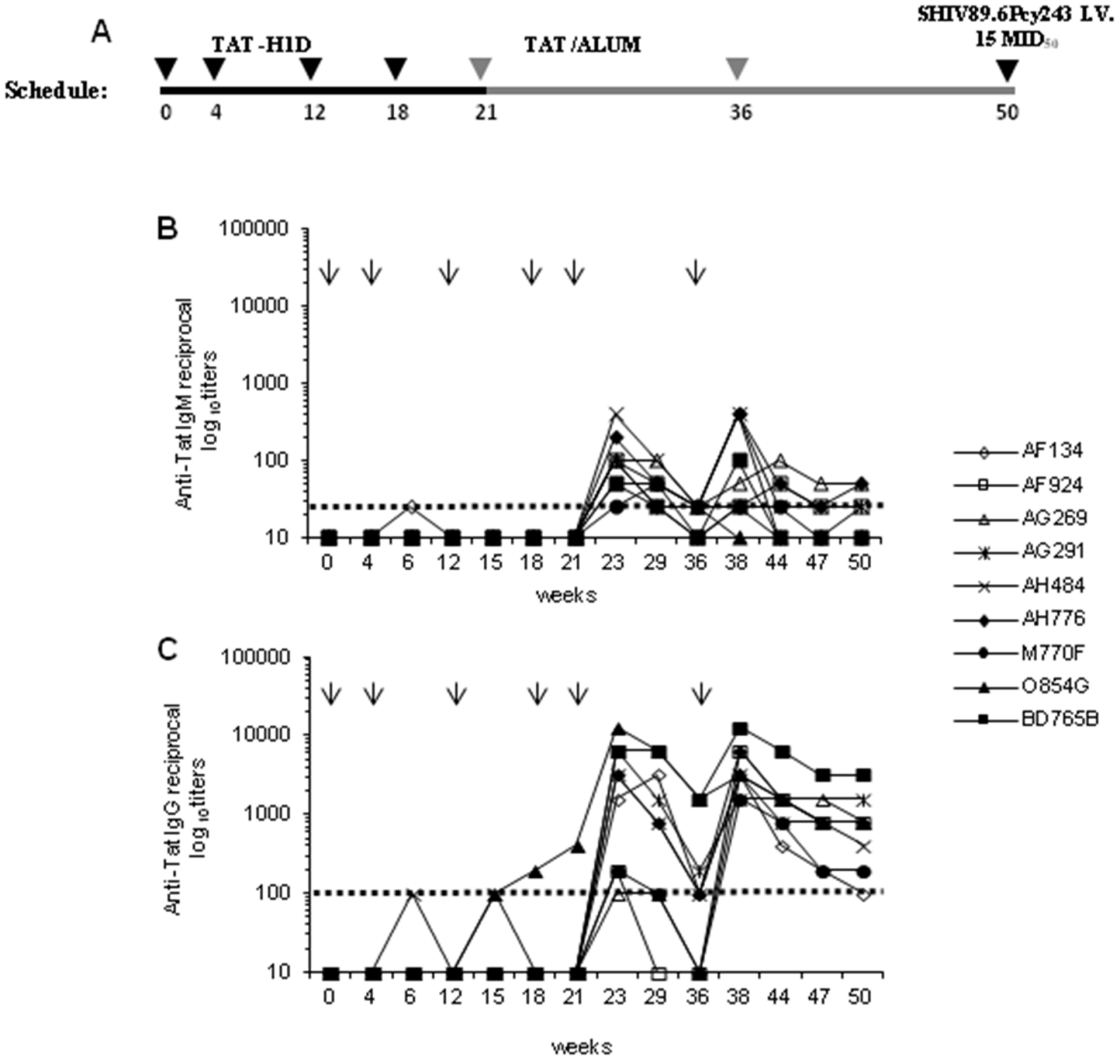
Schedule of immunization and anti-Tat antibody responses in cynomolgus monkeys vaccinated with Tat/H1D and Tat/Alum. (**A**) Nine monkeys were injected intramuscularly with Tat/H1D microspheres at weeks 0, 4, 12 and 18, and boosted subcutaneously with the Tat in Alum at weeks 21 and 36, respectively. Nine control monkeys were primed with H1D alone boosted with Alum alone. (**B**) IgM and (**C**) IgG antibody titers in vaccinated monkeys. The arrows on the top of the each panel indicate the time at which the Tat/H1D, the Tat/Alum or H1D and Alum alone were given. The dashed lines indicate the cut-off values (samples showing titers <1∶25 for IgM and <1∶100 for IgG were scored as negative).

Nine monkeys (AF134, AF924, AG269, AG291, AH484, AH776, M770F, O854G, BD765B) were immunized i.m. at weeks 0, 4, 12 and 18 with the Tat (10 µg)/H1D (60 µg) formulation and boosted subcutaneously (s.c.) at weeks 21 and 36 with Tat (10 µg) in the presence of Alum (Fig. 1A). The dose of Tat (10 µg) was chosen based on previous immunization studies in monkeys (22) while the Tat/H1D ratio used was chosen because it gives an adsorption efficiency of 100% as determined previously (23, 25). Nine control monkeys (AC601, AI075, AF942, AG249, AG347, AG934, AC032, AC739, AC921) received the H1D microspheres and Alum alone according to the same schedule and routes of administration. At week 50 all monkeys were challenged intravenously (i.v.) with 15 MID_50_ of the highly pathogenic SHIV89.6P_cy243_, grown and titrated in cynomolgus monkeys [34].

### Analysis of humoral responses

Antibody responses against HIV-1 Tat and Env (IgM, IgG) proteins were measured in plasma of immunized and control monkeys by enzyme-linked immunosorbent assay (ELISA) using 96-well immunoplates (Nunc-immunoplate MaxiSorp Nunc, Kampstrup, Denmark) previously coated with 100 ng/well of Tat or Env proteins, as described [35]. The cut-off values of the assays were defined as the mean OD_405_ (+3 SD) of a panel of pre-immunization samples and of plasma from naïve cynomolgus monkeys.

Peptides (15-mers overlapping by 10 aa) spanning the 1–90 aa of HIV-1 Tat isolate BH10 (Gen Bank accession number M15654) protein sequence were synthetized by UFPeptides (Ferrara, Italy) and used for IgG epitope mapping. Epitope mapping was performed upon coating the plates with 250 ng/well of single 15-mers Tat peptides, as described [35].

Anti-Tat IgG isotypes were determined on microtiter plates coated with the Tat protein, as described above, and incubated with plasma (duplicate wells) of vaccinated and control monkeys, serially diluted in PBS containing 0.05% of Tween-20, for 1 hour at room temperature. Following extensive washing, the plates were incubated with affinity purified sheep mAb (100 µl/well) directed against human IgG1 (diluted 1∶3,400), IgG2 (diluted 1∶3,400), IgG3 (diluted 1∶2,400), or IgG4 (diluted 1∶3,000) (AU006, AU007A, U008, AU009, respectively; The Binding Site Group Ltd, Birmingham, United Kingdom) for 1 hour at room temperature. After washing, the plates were then incubated with 100 µl/well of goat anti-sheep IgG horseradish peroxidase (HRP)-conjugated (diluted 1∶60,000) (A130-101P, Bethyl Laboratories Inc. Montgomery, TX, USA) for 45 minutes at room temperature. Finally, after addition of 100 µl/well of ABTS substrate (Roche, Mannheim, Germany), the absorbance value of each well was read with a spectrophotometer at 405 nm wavelength. The cut-off value for each IgG subclass was calculated as the mean OD_405_ (+4 SD) of a panel of pre-immunization samples and plasma from naïve cynomolgus monkeys. Known positive and negative monkey samples were included as internal controls in each plate of the different ELISA.

### Lymphoproliferative responses

PBMCs (2×10^5^/well) were seeded in 96-well microtiter plates in 200 µl of culture medium and incubated at 37°C with: i) medium alone (negative control); ii) PHA (2 µg/ml) (positive control), or iii) a pool of all Tat peptides (2 µg/ml of each peptide), in triplicate wells. After 5 days, cells were pulsed with 1 µCi/well of [^3^H] thymidine (Amersham, Buckinshamshire, United Kingdom) and harvested 18 hours later onto filter paper using a cell harvester (Tomtec, Orange, CT, USA). The incorporated radioactivity was measured in a β-counter. The stimulation index (S.I.) was determined as the ratio between the mean counts/minute of antigen-stimulated wells and the mean counts/minute of the same cellular sample incubated with medium alone (negative control). Stimulation indices (S.I.) ≥3 were considered positive.

### Analysis of T lymphocytes subsets

CD4+ and CD8+ T cell subsets were analyzed in citrated peripheral blood (100 µl) using anti-CD3^FITC^ (clone FN-18 Biosource, InVitrogen, Camarillo, CA, USA), anti-CD8^PerCP^ and anti-CD4^PE^ (clone SK-1, clone SK-3 respectively, Becton Dickinson Immunocytometry System, San Josè, CA, USA) mAb (20 µl each) for 30 minutes at 4°C. After washing with PBS supplemented with 2% FBS (Hyclone), cells were fixed in PBS containing 1% (w/v) paraformaldehyde and analyzed by cytofluorimetry (FACScan, Becton Dickinson Immunocytometry). Isotype-matched murine immunoglobulins conjugated with the different fluorochromes were used as controls. Absolute cell numbers were calculated from the blood cell counts.

### ELISpot assays

Enzyme-linked immunospot (Elispot) assays were performed for Th1 (IFN-γ, IL-2) and Th2 (IL-4) cytokines using commercially available monkey’s IFN-γ, IL-2 and IL-4 ELISpot reagents (Mabtech AB, Nacka Strand, Sweden), as previously described [26]. Briefly, PBMCs (2×10^5^) were seeded in 96-well multiscreen plates (Millipore Corporation, Billerica MA, USA), previously coated with mAb against simian IFN-γ, IL-2 or IL-4, in the presence of: i) medium alone (negative control), ii) PHA (2 µg/ml) (positive control for cytokine release) or iii) a pool of all Tat peptides (2 µg/ml of each peptide), in duplicate wells. Spots were counted using an automated ELISpot reader (A.EL.VIS, Hannover, Germany). Results are expressed as number of spot forming cells (SFC)/10^6^ cells after subtraction of the background (the number of SFC/10^6^ cells detected in the unstimulated sample). The cut-off values were calculated as the mean number of SFC/10^6^ cells (+2 SD) determined in PBMC cultures from naïve monkeys stimulated with the Tat peptides upon subtraction of the background. Fold-increase over the background was also calculated. According to both criteria, samples yielding for IFN-γ ≥80 SFC/10^6^ cells and a fold increase ≥2.5, for IL-2≥30 SFC/10^6^ cells and a fold increase ≥3, for IL-4≥20 SFC/10^6^ cells and a fold increase ≥2.5, were considered positive.

### Determination of viral loads

Viral RNA in plasma was quantitated in cell-free plasma by a highly sensitive real time RT-PCR assay with a threshold limit of 50 RNA Eq/mL, as described [2]. Proviral copies were quantitated in DNA samples extracted from 400 µl of whole citrated blood using the TaqMan real-time PCR with a threshold limit of detection of 10 copies/µg DNA. Probe and primers specifically amplifying a region of 71 bp within the *gag* sequence of SIVmac251 (GI:334657) were designed and thermal cycling conditions were used as previously described [36]. Samples were analyzed in duplicate by DNA-PCR in a total volume of 25 µl of a mixture containing 400 ng of DNA, 12.5 µl of PCR master mix (Applied Biosystems, Carlshad, CA, USA), 900 nM of each primer and 180 nM of probe.

### MHC analisis of cynomlgus macaques

MHC class I*A* and I*B* and class II haplotypes were determined by microsatellite PCR with resolution of recombinant class I*B* haplotypes by allele-specific PCR as previously described [37], [38].

### Statistical analysis

RNA plasma viremia, DNA proviral loads and CD4**^+^** T cell counts were evaluated in the acute (up to week 4), the post-acute (weeks 8–12) and the chronic (weeks 22–74) phase of the infection. The rate of the CD4^+^ T cell decline in acute infection was calculated on the last CD4**^+^** T cell count prior to challenge (baseline). In each phase of infection, Tat**-**vaccinated versus control monkeys were separately analyzed with a regression model for correlated data. In this analysis each animal was considered as an independent sample, and each measure within the same animal was considered as a dependent sample. The same model was used to evaluate the relationship between anti-Tat IgG antibodies and viral loads or CD4**^+^** T cells, respectively, in each treatment group. The smoothed curve of viral loads and CD4**^+^** T cells were estimated using a smooth local regression (LOESS), with a quadratic fit and a span of 0.5. Statistical analyses and data processing were performed using SAS® software (SAS Institute, Cary, NC, USA). All statistical tests were performed at a two-sided 5% significance level. Nonparametric Spearman rank correlation analysis was used (GrapPad InStat vers 3.05 software, San Diego, Ca, USA) to evaluate the correlation between the IgG subclasses and their association with T-cell responses and the outcome of viral infection. Similarly, GrapPad InStat was used to calculate the mean of the OD values, the standard error of means (SEM), and to evaluate the differences of epitope reactivities between controllers and viremic macaques (2-tailed unpaired T test).

## Results

### Administration of H1D microspheres with or without Tat is safe in cynomolgus monkeys

Nine adult male cynomolgus monkeys received the Tat protein formulated with the H1D microspheres (priming, i.m.) or with Alum (boost, s.c.), while other nine control monkeys were injected i.m. with the H1D microspheres and s.c. with Alum alone (Fig. 1A). Safety was evaluated by monitoring the weight and hematological parameters of vaccinees and control monkeys during the all course of immunization and compared to a group of naïve cynos (n = 21) matched for sex, age and weight within the same time frame. Overall, no major local reactions were recorded after immunizations and all parameters evaluated were in the normal range at all times, indicating that the H1D microspheres alone or formulated with Tat are safe (Table S1), as previously reported in mice [23], [25].

### Tat/H1D priming and Tat/Alum boosting immunization elicits broad and long-lasting humoral and cellular anti-Tat immunity

Anti-Tat IgM (Fig. 1B) and IgG (Fig. 1C) antibody responses were detected at low titers only in a few monkeys after Tat/H1D immunization (up to week 21), whereas they promptly increased to high titers in all vaccinees after each of the boosts with Tat/Alum, given at weeks 21 and 36. This kinetics resembles the pattern of humoral responses reported in monkeys vaccinated with a DNA vector [36] and indicates that the Tat/H1D vaccine had primed the B cell arm of immunity, whereas the Tat/Alum boosting strengthened such responses. None of the control monkeys developed anti-Tat antibody responses (data not shown). At the time of challenge (week 50), anti-Tat IgM were either absent in 4 monkeys (AF134, M770F, O854G, BD765B) or present at very low titers (range 1∶25–1∶50) in 5 (AF924, AG269, AG291, AH484, AH776) out of 9 vaccinated monkeys, whereas IgG antibody were present at higher titers in all vaccinees (range 1∶100–1∶3,200), indicating that the prime/boost immunization protocol induced high and durable Tat-specific antibody responses.

**Figure 2:**
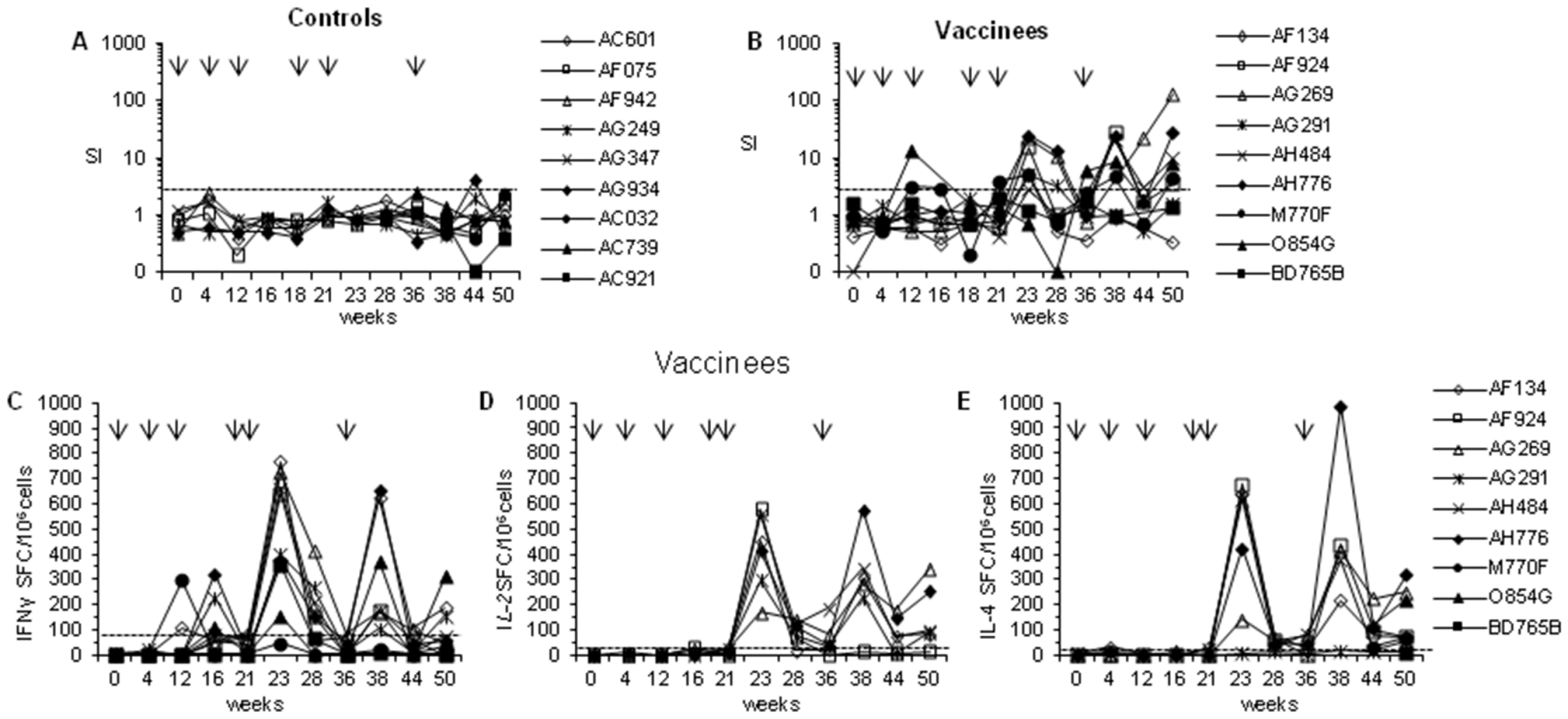
Lymphoproliferative responses and frequency of cells producing IFN-γ, IL-2 or IL-4 during vaccination. The proliferative responses of (**A**) control and vaccinated (**B)** monkeys are reported as Stimulation Index (S.I). The dotted line indicated the cut-off of the assay. All samples showing a S.I. >3,0 were scored as reactive. In the lower panels the number of Spot Forming Cells (SFC/10^6^ PBMCs) of vaccinated macaques upon in vitro stimulation with Tat peptide pool are reported for the production of (**C**) IFN-γ, (**D**) IL-2 and (**E**) IL-4. None of the control macaques exhibited T cell responses and therefore the data are not presented. Arrows on the top of each panel indicate the time at which the vaccine antigen was given. The dashed line represents the cut-off (SFC/10^6^ PBMCs) of the assay. However, as described in the Material and Methods, were considered positive only samples yielding for IFN-γ ≥80 SFC/10^6^ cells and a fold increase ≥2.5; for IL-2≥30 SFC/10^6^ cells and a fold increase ≥3; for IL-4≥20 SFC/10^6^ cells and a fold increase ≥2.5.

Next, the ability of this vaccine regimen to stimulate cell-mediated immune responses was evaluated by measuring lymphoproliferation and the frequency of PBMCs producing Th1-type (IFN-γ, IL-2) and Th2-type (IL-4) cytokines upon *in vitro* stimulation with Tat peptides. With a kinetics similar to that observed for anti-Tat antibody production, proliferative responses against Tat were undetectable or very low after priming with Tat/H1D and rapidly boosted by Tat/Alum inoculations (Fig. 2B). Indeed, high S.I. values were readily measured two weeks after each Tat/Alum boost (S.I. range: 4.8–27.8 and 3.4–128.9, at week 23 and 38, respectively) and still detectable at the time of challenge (week 50) in 6 (AF924, AG269, AH484, AH776, M770F, O854G) out of 9 vaccinated monkeys, while antigen-specific proliferation was not observed in control monkeys (week 23, S.I. range 0.8–1.2; week 38, S.I. range 0.4–2.3) (Fig. 2B).

Of interest, 5 (AF134, AG291, AH776, M770F, O854G) out of 9 vaccinated monkeys exhibited IFN-γ ELISpot responses at earlier time points (weeks 12 or 16) during the priming (range: 110–325 SFC/10^6^ cells) (Fig. 2C). These responses peaked at week 23 and 38, i.e., two weeks after each Tat/Alum boost, when they became detectable in all but one (M770F) vaccinated monkeys but then declined in spite of the second boost. At the time of challenge (week 50) only 3 vaccinated monkeys (AF134, AG291, O854G) presented IFN-γ responses (range: 155–312 SFC/10^6^ cells). Similarly, IL-2 (Fig. 2D) and IL-4 (Fig. 2E) responses became detectable in all macaques at weeks 23 and 38, after the two boosts, with the exception of monkey AG291 for which IL-4 responses were never detected. However, in contrast to IFN-γ, IL-2 and IL-4 responses were more durable and still detectable up to week 50 in most vaccinees (5 out of 6 for IL-2, AF134, AG269, AG291, AH484, AH776; range: 93–343 SFC/10^6^ cells; 7 out of 9 for IL-4, AF134, AF924, AG269, AH484, AH776, M770F, O854G; range: 58–320 SFC/10^6^ cells). No Tat-specific cytokine production was ever detected in control monkeys (data not shown). Altogether these data indicate that injection of the Tat/H1D primed broad T cell responses, which were expanded by the Tat/Alum boosts, as indicated by the detection of Th1- and Th2-type memory T cells.

### Tat/H1D priming and Tat/Alum boosting immunization significantly controls plasma viremia, reduces CD4^+^ T cell loss and restores CD4^+^ T cell counts upon intravenous challenge with SHIV89.6P_cy243_

**Figure 3:**
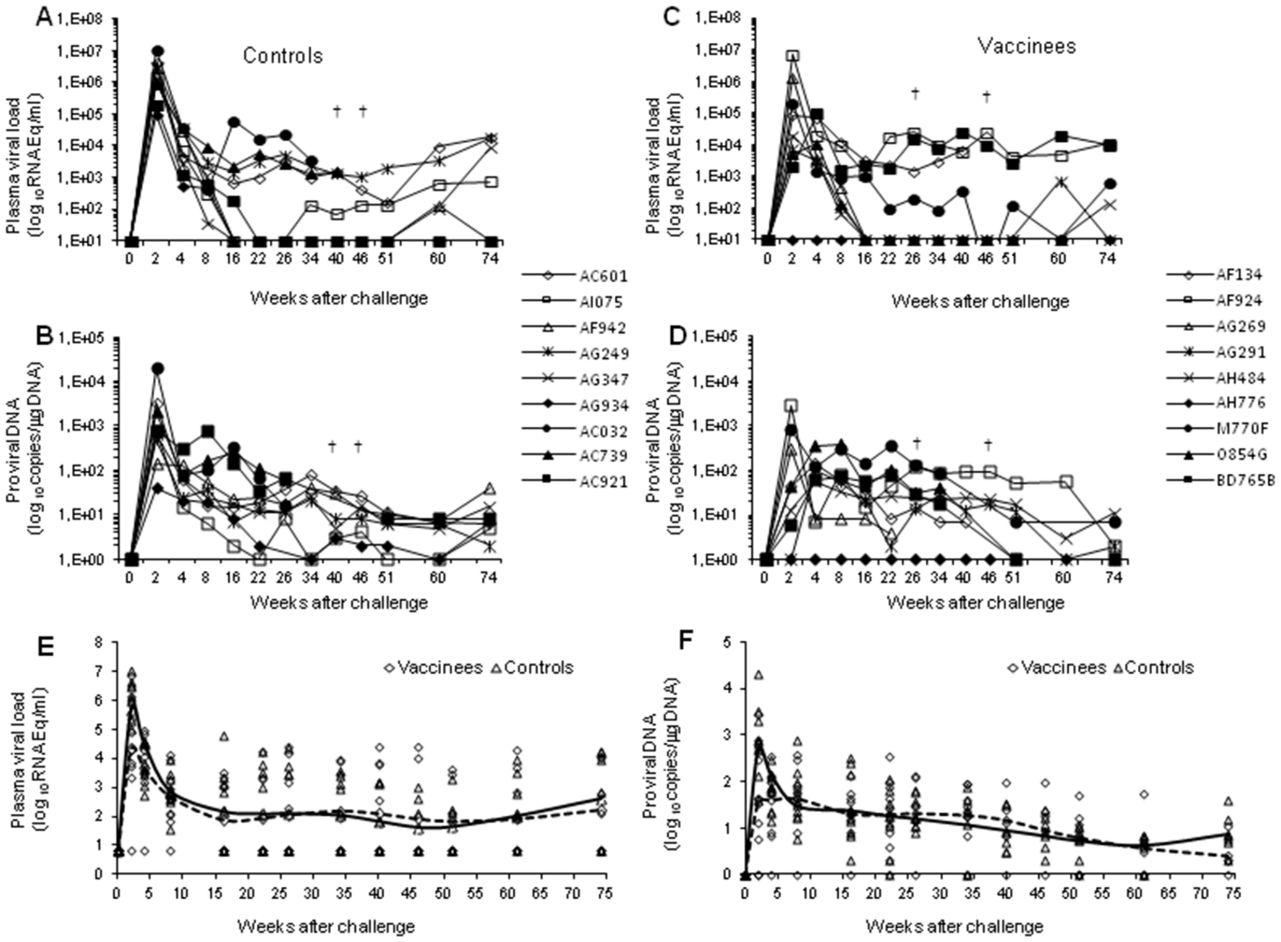
Quantitation of viral load upon intravenous virus challenge with SHIV89.6P_cy243_ in control and vaccinated monkeys. In the left panels are reported the (**A**) plasma viremia and (**B**) the proviral DNA of control macaques. In the right panels are reported the (**C**) plasma viremia and the (**D**) proviral DNA of vaccinated macaques. In the bottom panels are indicated the trend line as a LOESS smoothed average of (**E**) plasma viral RNA and (**F**) proviral DNA of vaccinated (dashed line) and control (continous line) macaques. Statistical analyses were performed according to the Regression model for correlated data (means with 95% confidence limits).

At week 50, all monkeys were inoculated intravenously with 15 MID_50_ of the highly pathogenic SHIV89.6P_cy243_ grown in cynos [34]. All control animals became viremic with a peak viremia (1×10^5^−1×10^7^ RNA Eq/ml) at week 2 after infection (Fig. 3A). Afterwards, from week 8 to week 74, RNA viral loads in plasma declined although remaining always detectable (range: 6.4×10−1.7×10^4^ RNA Eq/ml), with the exception of monkeys AC921 and AG934 that experienced the lowest peak viremia, which then fell below the threshold limit of detection of the assay (50 RNA Eq/ml). In agreement with this kinetics, the proviral loads peaked two weeks after challenge in all control monkeys and remained detectable (range: 39–20,261 proviral copies/µg DNA) up to week 74, irrespective of the viremia levels (Fig. 3B). Two control monkeys (AC032, AC739) died at weeks 40 and 46 after challenge, respectively.

In contrast, in the vaccinated group, one animal (AH776) remained persistently aviremic, 6 (AF134, AF924, AG269, AG291, AH484, M770F) became viremic with a peak (7.1×10^3^–6,1×10^6^ RNA Eq/ml) at week 2 after challenge, while the remaining two cynos, O854G and BD765B, exhibited a delayed viral RNA peak (1.1−9.4×10^4^ RNA Eq/ml) at week 4 (Fig. 3C). Of the 8 viremic vaccinees, 3 remained viremic (AF134, AF924, BD765B), 3 (AF291, AH484, O854G) became aviremic with sporadic bleeps of viremia (1.35–7.2×10^2^ RNA Eq/ml) just above the threshold limit of detection of the assay. The remaining 2 vaccinees (AG269 and AF134) died at weeks 26 and 46 after challenge, respectively. Also the kinetics of proviral loads was different, as compared to controls (Fig. 3D). In fact, the proviral load peaked at week 2 only in 3 vaccinees (AF924, AG269, M770F) (range 288–2,875 proviral copies/µg DNA), whereas in other 5 monkeys (AF134, AG291, AH484, O854G, BD765B) the peak was reached at weeks 4–8 after challenge (range: 57–379 proviral copies/µg DNA). Of note, proviral DNA was never detected in the vaccinated and aviremic monkey AH776.

Viral RNA (Fig. 3E) and proviral DNA (Fig. 3F) levels of vaccinees and controls were compared during the three different phases of infection, including the acute phase (mean of weeks 2–4), the post-acute phase (mean of week 8–16), and the chronic phase (mean of weeks 22–74). Remarkably, viral RNA and DNA loads were significantly lower in the acute phase of infection in vaccinees as compared to control monkeys (p = 0.0072 and p = 0.0417, respectively). Although these differences were no longer appreciable in the post-acute and chronic phases of infection, two different patterns of viral replication dynamics became apparent in the vaccinated monkeys during the chronic phase (weeks 22–74). In fact, out of the 8 macaques with an extended follow-up, 4 monkeys displayed a persistently detectable (27/32 samples, 93.1%) viremia (range: <50−1.6×10^5^ RNA Eq/ml), whereas in the remaining 4 monkeys viremia was sporadic (2/30 samples, 6,6%) and much lower (range: <50−1.8×10^2^ RNA Eq/ml) (Table 1). Similarly, proviral DNA was higher (mean: 58.6±78.3 copies/µg DNA) and more frequently detectable (19/21 samples, 90.8%) in the viremic vaccinees as compared to the aviremic ones (16/25 samples, 64%; mean: 15.72±21.03 copies/µg DNA) (data not shown). These differences were statistically significant (p<0.0001 for viremia and p<0.017 for proviral load) and, based on these virological data, we arbitrarily termed the former as “viremics” and the latter as “controllers” (Table 1).

**Table 1:** Summary of the virological and immunological status of “viremic” and “controller” vaccinated monkeys during the chronic phase of infection (weeks 22–74).

Vaccinated monkeys^a^	Virological status b (RNA Eq/ml, range)	CD4^+^ T cell counts^c^(range)	Anti-Tat antibody titers^d^ (range)	Clinical Benefit
AF134	V (2.3×10^3^–69×10^3^)	20−40	<100	No
AF924	V (4.1×10^3^–2,5×10^4^)	111−231	<100–200	No
M770F	V (<50−3.6×10^2^)^e^	1300–2356	<100–200	Yes
BD765B	V (8.3×10^2^–1.6×10^5^)	18−69	200–800	No
	p<0.00001	p = 0.0004	p<0.0001	
AH484	C (<50−1.3×10^2^)	876–1420	100–400	Yes
AH776	C (<50)	487−2399	100–800	Yes
AG291	C (<50)	1130−2270	200–400	Yes
O854G	C (<50–1.8×10^2^)	880−2560	200–800	Yes

a Monkey AG269 died at week 26 after challenge and was excluded from the analysis. Monkey AF134 died at week 46 after challenge.

b According to their virological status during chronic infection, vaccinated monkeys were grouped as viremic (V) and Controllers (C). The numbers in parenthesis indicate the range (minimum-maximum value) of plasma viral RNA (Eq/ml). The p value indicates the statistical difference between the two groups.

c Numbers in parenthesis indicate the range (minimum-maximum value) of CD4**^+^** T cell counts/mmc. The p value indicates the statistical difference between the two groups.

d Numbers in parenthesis indicate the range (minimum-maximum value) of anti-Tat IgG antibody titers. The p value indicates the statistical difference between the two groups.

e This monkey was negative for plasma viremia at 2 out 9 time points during the chronic infection.

**Figure 4:**
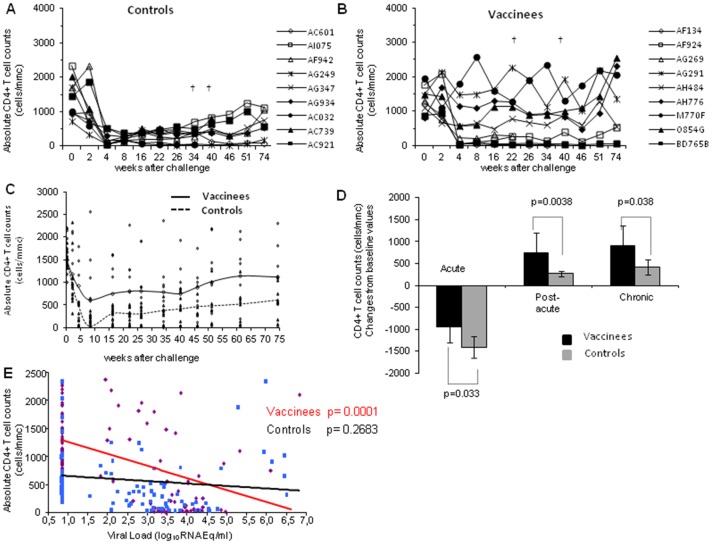
Absolute numbers of CD4^+^ T cells following challenge with SHIV89.6P_cy243_. The absolute CD4^+^ T cell counts are reported for (**A**) control and (**B**) vaccinated monkeys. In the left middle panel (**C**) the trend line as a LOESS smoothed average of the values of control (dashed line) and vaccinated (continous line) monkeys is shown. (**D**) Statistical analysis of the changes from baseline of CD4^+^ T cell counts in vaccinated and control macaques during the acute, post-acute and chronic phases of infection. The numbers within the panel indicate the level of statistically significant differences. (**E**) Analysis of the correlation of plasma viremia and CD4^+^ T cells counts in vaccinated (continuous red line) and control (black line) monkeys is reported.

Overall, the different outcomes and kinetics of infection reported above were confirmed by the CD4^+^ T cell counts. In fact, all control macaques suffered a profound CD4^+^ T cell loss (<518 CD4^+^/mmc) in the acute phase of infection (Fig. 4A), due to the robust viral replication, and two of them (AC032 and AC739) died at weeks 40 and 46, respectively. The depletion (<518 CD4^+^/mmc) persisted in the post-acute and chronic phases of infection with the exception of monkeys AI075, which experienced a stable, although partial, CD4^+^ T cell recovery (699−1,239 CD4^+^/mmc). In contrast, only 4 (AF134, AF924, AG269, BD765B) out of the 9 vaccinated macaques exhibited CD4^+^ T cell counts below 500 cells/mmc (range: 20–217 cells/mmc) at week 4 after challenge (Fig. 4B). Of them, two underwent a severe CD4^+^ T cells depletion (AF134, 97%; AG269, 80%) and died at weeks 26 and 46, respectively. Of the remaining 5 vaccinees, 3 (AG291, AH776, M770F) did not experience any loss of CD4^+^ T cell during the acute, post-acute and chronic phase of infection, maintaining CD4^+^ T cell counts comparable to the baseline values (range 1,104−2,309 cells/mmc). Macaques O854G and AH484, which during the acute infection had experienced a limited loss of CD4^+^ T cells (571 and 561 CD4^+^ T cell/mmc, respectively), either greatly regained (O854G) or maintained (AH484) their CD4^+^ T cell counts. Interestingly, vaccinees M770F and BD765B, which had a high peak viremia (2.1×10^5^ and 9.4×10^4^ RNA Eq/ml, respectively) and remained viremic in the chronic phase of infection, exhibited a very divergent dynamics with regard to the CD4^+^ T cells counts. In fact, monkey M770F returned to almost normal CD4^+^ T cell level by week 4 after challenge, while monkey BD765B did not (Fig. 4B).

Overall, the divergent dynamics of CD4+ T cell counts in controls and vaccinees is indicated in Fig. 4C. Accordingly, when CD4+ T cell counts and plasma viral loads of vaccinated and control monkeys were compared in the different phases of infection, the beneficial effects of Tat/H1D/Alum immunization was evident (Fig. 4D). In fact, the degree of CD4^+^ T cell depletion (acute infection) or recovery (post-acute and chronic phases) in vaccinated macaques was significantly different from that observed in control monkeys (p = 0.033, p = 0.0038 and p = 0.039, respectively). Notably, the level of CD4^+^ T cells in vaccinated macaques significantly correlated with lower levels of viral RNA in plasma (p<0.0001) (Fig. 4E).

### Cellular and humoral immune responses after virus challenge

After challenge, Tat-specific T-cell responses in control macaques were either undetectable or sporadically detected for IFN-γ at weeks 16, 22 and 34 and for IL-2 at week 40 (Fig. S1 A–D). Proliferative responses, as well as IFN-γ, IL-2 and IL-4 ELISpot responses were not boosted after challenge in the vaccinated monkeys (Fig. S1 E–H), although it is worth noting that in the acute, post-acute and early chronic phases of infection (weeks 4, 8–22) some macaques exhibited T cell responses, in particular IL-2 and IL-4, resembling an anamnestic response to viral infection.

**Figure 5:**
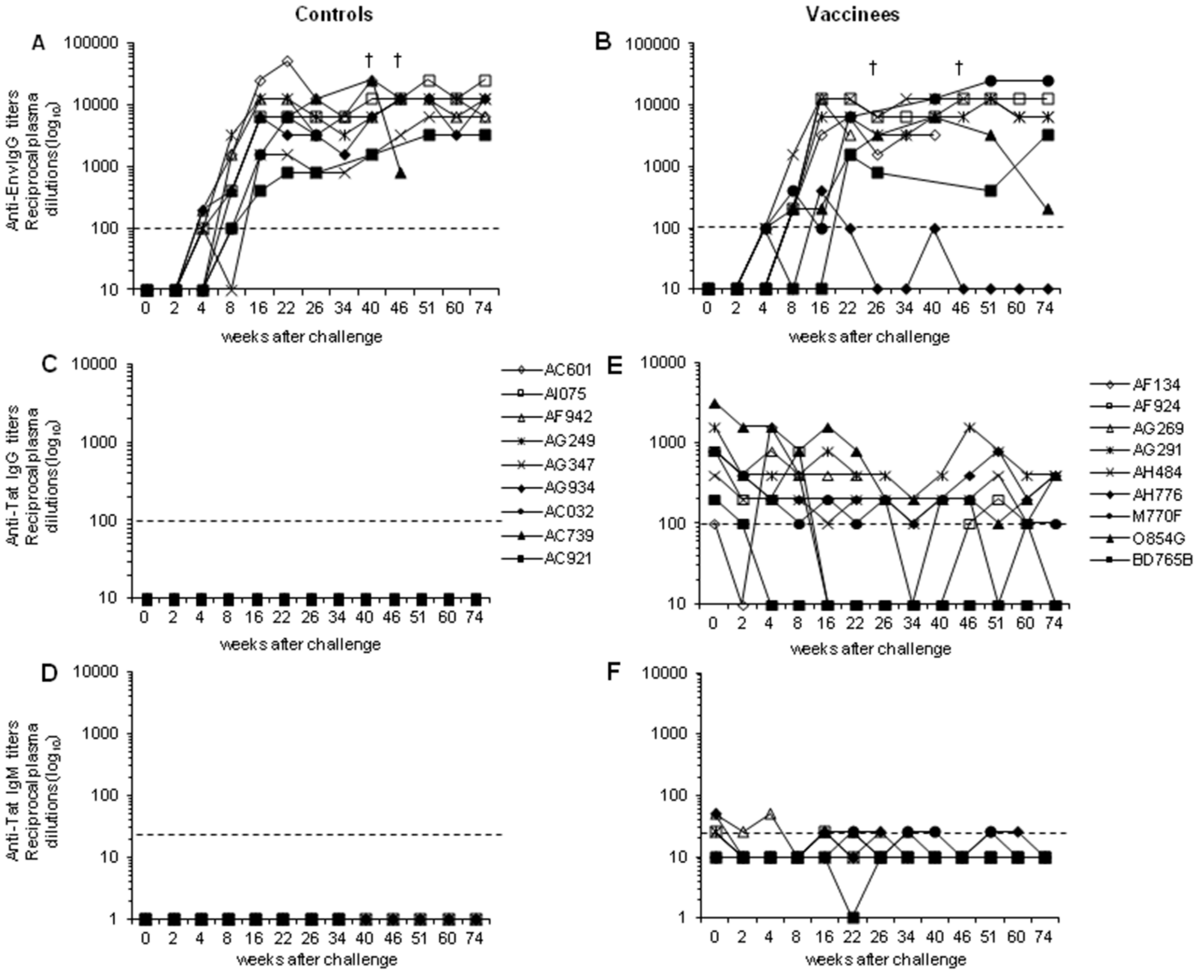
Antibody responses against HIV-1 Env and Tat proteins after challenge with SHIV89.6P_cy243_. Anti-Env IgG antibody titers were determined in plasma of control (**A**) and vaccinated (**B**) macaques. In the middle panels are reported the anti-Tat IgG antibody titers in (**C**) controls and (**D**) vaccinees. In the bottom panels the anti-Tat IgM antibody titers in (**E**) controls and (**F**) vaccinees are indicated. The dashed lines indicate the cut-off values (samples showing titers <1∶25 for IgM and <1∶100 for IgG were scored as negative).

Upon challenge, all control monkeys seroconverted, and anti-Env IgG antibody titers increased reaching a plateau in the chronic phase of infection (Fig. 5A). Similarly, all but one vaccinated monkey (AH776) seroconverted by week 8 (Fig. 5B), and 5 of them (AF134, AG291, AH484, MF770, BD765B) showed increased anti-Env IgG antibody titers afterwards, reaching levels comparable to those observed in the controls. Interestingly, monkey AH776, which exhibited undetectable levels of plasma viremia and proviral load, clearly seroconverted by week 16, showing thereafter lower titers of anti-Env antibodies as compared to the other vaccinees. Among the control macaques, anti-Tat IgG (Fig. 5C) and IgM (Fig. 5D) antibodies were never detectable after challenge. As already observed for T cell responses, vaccinees did not exhibit anti-Tat IgG (Fig. 5E) and IgM (Fig. 5F) anamnestic responses. In particular, anti-Tat IgM antibodies declined soon after challenge, becoming either undetectable or barely detectable in a few monkeys (titer ≤1∶50).

### Pre-challenge antibody responses to Tat and outcome of infection

**Figure 6:**
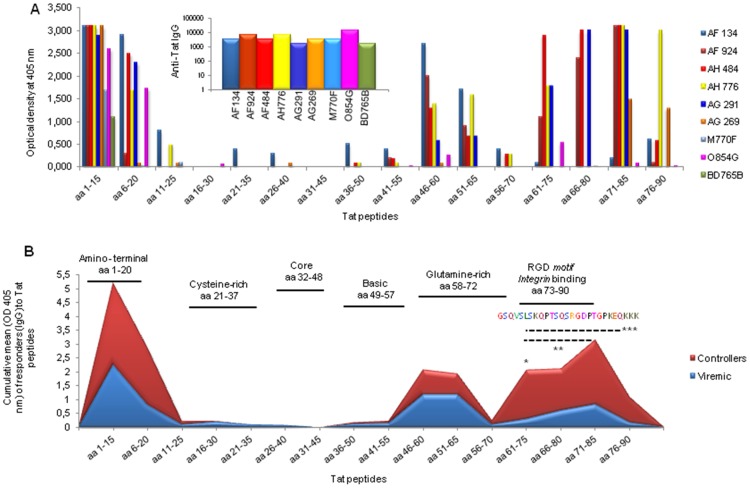
Epitope mapping and virological outcome. (A) Plasma anti-Tat IgG responses to single Tat peptide were determined by ELISA at week 38 after the first immunization. To map specific responses, individual 15mer peptides (overlapping by 10 aa) spanning the aa 1–90 of Tat were used. The histogram inserted within the graph represents the anti-Tat IgG titers of vaccinated monkeys at week 38. (**B**) In the area plot graph is reported the cumulative means of the O.D. values of vaccinees grouped according to their virological status (controllers **in red** and viremics **in blue**). The dashed line with one, two or three asterisks represents the statistical analyses (Mann-Whitney test or unpaired t-test; GrapPad InStat vers 3.05 software, San Diego, Ca, USA) performed by comparing the epitope reactivity to the indicated Tat domains of controllers versus viremic macaques. These analyses were performed either comparing the reactivities to each single peptide (aa 61–75; * p = 0,037) or to grouped peptides (aa 61–90, *** p = 0,0084 and aa 61–85, ** p = 0,014). On the top of the graph are reported the Tat aa sequence and the associated Tat functional domains. The colored aa sequence represents the region against which anti-Tat antibodies from controllers but not viremic animals are directed.

As described above, vaccinated monkeys were grouped in controllers and viremics according to the patterns of viral replication dynamics in the chronic phase of the infection (Table 1). Of interest, controllers had persistently measurable anti-Tat antibodies in the post-acute and chronic infection, whereas viremics did not. When also the CD4 T cell counts were considered, it became apparent that controllers exhibited during the chronic phase of the infection a significant reduction of viral loads (p<0.0001), high and durable anti-Tat IgG antibody titers (p<0.0001) and CD4^+^ T cell counts (p = 0.0004), as compared to viremics (Table 1), suggesting a role for anti Tat antibody responses in the observed control of viral replication in vaccinees. To gain further insights, we analyzed the epitope specificity of IgG antibodies after the last Tat/Alum boost (week 38). In this respect, all vaccinated macaques showed reactivity directed in particular against peptides encompassing the Tat N-terminus (aa 1–20). The next most recognized domains was aa 46–60 (7 out of 9 monkeys), followed by the regions encompassed by aa 51–65, aa 61–75 and aa 71–85, which were recognized by the plasma of 6 out of 9 monkeys, while the aa 76–90 domain was recognized by 5 vaccinees (Fig. 6A). Only a minority of vaccinees had detectable antibodies against the remaining Tat domains: 4 monkeys reacted with aa 36–55, whereas only 3 macaques reacted with the aa 56–70 or aa 66–80 domain (Fig. 6A). As the anti-Tat IgG titers were similar in all monkeys (range 1∶1,600/12,600), the observed epitope specificity conceivably reflects qualitatively different responses (Fig. 6A) in vaccinated animals experiencing different challenge outcome. For instance, monkey BD765B (vaccinated and viremic), in spite of having anti-Tat IgG titers 1∶12,600, did not show reactivity to the Tat peptides encompassing aa 61–85. In contrast, the AH484 (vaccinated and controller), which exhibited a lower anti-Tat Ab response, did react to the 61–85 Tat peptides (OD range 2.9–3.1).

Intriguingly, when vaccinated macaques were grouped according to their virological status (controllers versus viremics), a number of interesting observations emerged (Fig. 6B). As determined by the O.D. readings, neither controllers nor viremics exhibited relevant responses to peptides covering the aa 11–55 domain of Tat. The viremics had a higher, although statistically not significant, reactivity against the aa 46–65 region of Tat (mean O.D. = 1.175, SEM = 0.453) than controllers (mean O.D. = 0.822, SEM = 0.198) (Fig. 6B). In contrast, controllers, as compared to viremic vaccinees, exhibited higher mean O.D. values directed against peptides encompassing the N-terminal region (aa 1–20; mean O.D. = 2.503, SEM = 0.197 for controllers; mean O.D. = 1.562, SEM = 0.484 for viremic), and against peptides spanning the C-terminal half of Tat (aa 56–90) which contains functionally relevant Tat domains (basic region, aa 48–57; glutamine-rich region, aa 58–72; exon 2, including the integrin binding-RGD domain of Tat, aa 73–86) (mean O.D. = 1.33, SEM = 0.306 for controllers; mean O.D. = 0.401, SEM = 0.191 for viremic animals) (Fig. 6B). Of note, the aviremic macaque AH776 was the only one vaccinee showing a high reactivity to both aa 71–85 and aa 76–90. When responses to aa 61–75, aa 66–80, aa 71–85 and aa 76–90 were grouped together, the difference between controllers and viremics became statistically significant (p = 0.010). Similarly, when responses to aa 61–85 and to aa 61–75 were analyzed, it turned out that controllers exhibited a higher and statistically different reactivity than viremic cynos (p = 0.016, p = 0.037). In the case of responses to aa 61–80, the difference between controller and viremic cynos, fell just below the threshold of statistical significance (p = 0.051). Taken together these results suggest that the sequences GSQTHQVSLSKQPTS within the aa 61–75 region and QSRGDPTGPK within the aa 71–85 region are relevant epitopes for the protective immunity.

**Figure 7:**
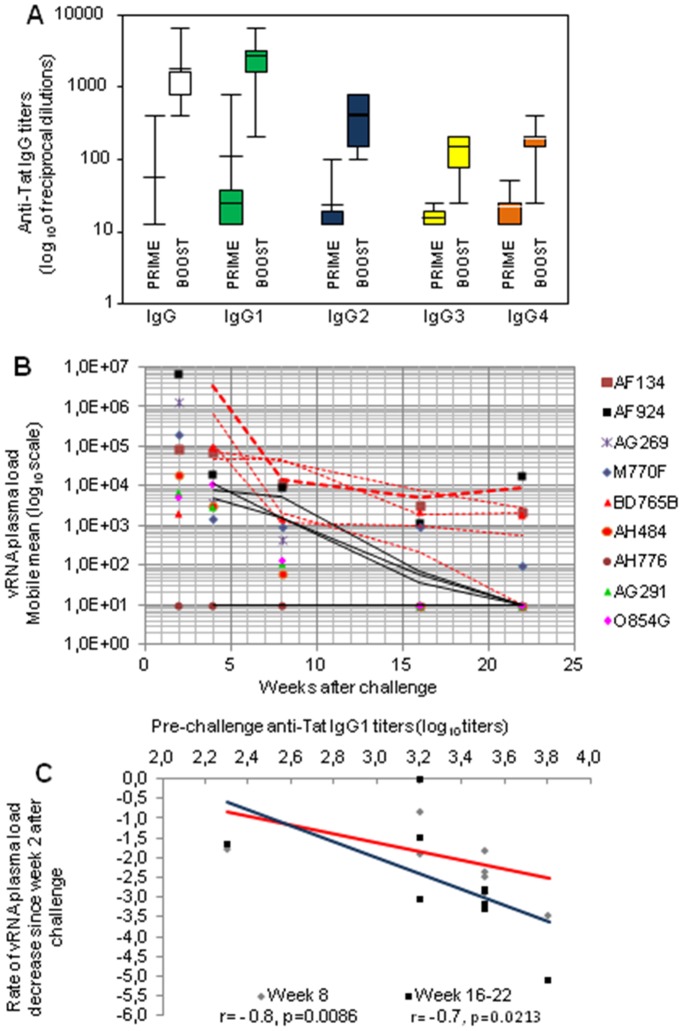
Anti-Tat IgG subclass profile before challenge and virological outcome. (**A**) The box-blot analysis represents the anti-Tat IgG and IgG subclass profile in plasma of cynomolgus macaques before (week 23) and after (week 44) the last Tat/Alum boost. For the anti-Tat IgG subclass profile, a cut off for each subclass was established based on the analyses of IgG subclasses in plasma of 30 naive cynomolgus macaques, as described in material and methods. (**B**) The rate of plasma viral load decline among vaccinees is indicated where the lines (dashed red lines for viremic; continous black lines for controllers) represent the mobile median starting from the first positive viremia sample during the acute phase of the infection. (**C**) The impact of anti-Tat IgG1 titers on the rate of vRNA decline during the post acute phase of the infection, as determined at week 44 during the immunization, is reported.

We next determined whether the profile of anti-Tat IgG subclasses induced by vaccination had a potential association with pre-challenge T-cell responses or could serve as a predictor of vaccine efficacy. Thus, anti-Tat IgG subclasses were evaluated at weeks 21 (3 weeks after the last Tat/H1D immunization) and 44 (7 weeks after the second Tat/Alum boost). It was immediately apparent that the Tat/Alum boost had markedly modulated the IgG subclass profile. In fact, after the Tat/Alum boost the IgG1 became the most represented isotype, followed by IgG2, and then by IgG3 and IgG4, which displayed comparable titers (Fig. 7A; Table S2). Thus, as already observed for T cell responses, the Tat/H1D/Alum vaccine elicited a balanced Th1/Th2 type of humoral responses.

While an association with other T cell responses was not observed, it is worth mentioning that in vaccinees anti-Tat IgG3 and IgG4 titers significantly correlated with the proliferative responses (r = 0.763, p = 0.036 and r = 0.732, p = 0.045 respectively; nonparametric Spearman rank analysis**)**. Later on, at week 50, anti-Tat IgG3 fell just below the threshold of significance (r = 0.683, p = 0.050), whereas anti-Tat IgG4 titers still remained associated to T cell proliferative responses (r = 0.821; p = 0.0083) (Table 2).

**Table 2:** Nonparametric Spearman rank analysis of the anti-Tat IgG subclass profile and anti-Tat T-cell responses (before challenge) in the vaccinated macaques.

	Time pre-challenge	IgG1	IgG2	IgG3	IgG4
Proliferative responses	week 38	r = 0.404 p = 0.326	r = 0.683 p = 0.069	r = 0.763 **p = 0.036**	r = 0.732 **p = 0.045**
	week 50	r = 0.469 p = 0.212	r = 0.489 p = 0.177	r = 0.683 **p = 0.050**	r = 0.821 **p = 0.0083**
IFN-γ	week 38	r = −0.568 p = 0.120	r = −0.275 p = 0.463	r = −7.7E–19 p>0.999	r = −0.211 p = 0.580
	week 50	r = −0.310 p = 0.410	r = −0.275 p = 0.463	r = −7.7E–19 p>0.999	r = −0.218 p = 0.580
IL-2	week 38	r = − 0.493 p = 0.355	r = −0.493 p = 0.355	r = 0.138 p = 0.802	r = 0.169 p = 0.713
	week 50	r = 1.205E–19 p>0.999	r = −0.277 p = 0.563	r = −0.130 p = 0.802	r = 0.338 p = 0.497
IL-4	week 38	r = −0.092 p = 0.919	r = 0.061 p = 0.919	r = 0.392 p = 0.419	r = 0.338 p = 0.497
	week 50	r = 0.102 p = 0.810	r = 0.228 p = 0.551	r = 0.452 p = 0.228	r = 0.522 p = 0.147

The impact of the IgG subclass profile on the virological outcome was also evaluated. Indeed, a significant direct association between anti-Tat IgG1 levels and viremia was detectable at week 2 after challenge (r = 0.74, p = 0.0238), but not at later times. In contrast, anti-Tat IgG3 and IgG4 titers exhibited a significant association with plasma viremia only at week 16 (r = −0.82; p = 0.0063 and r = −0.75; p = 0.0204 respectively; nonparametric Spearman rank analysis).

Finally, the impact of the anti-Tat IgG profile on the decline of viremia was analyzed. In this case the rate of decline was calculated starting from the viremia (mobile mean) measured at week 2. Vaccinees, although to a different degree, exhibited a rapid decline of plasma viral loads after the acute phase of infection (Fig. 7B). Pre-challenge anti-Tat IgG1 titers correlated with the rate of plasma viremia reduction in the post-acute phase of infection (r = −0.8 p = 0.0086 at week 8 and r = −0.7 p = 0.021 at weeks 16 and 22) (Fig. 7C). A similar trend was also observed in the post-acute phase of the infection for anti-Tat IgG4 (r = −0.66 p = 0.051 at weeks 8, 16 and 22).

To gain insights on the contribution of host MHC genotype to the outcome of the infection, the MHC class I and II haplotypes of all macaques included in this study were determined. Six haplotypes (M1 to M6), or simple recombinants thereof, were detected, with M1 and M3 being the most represented haplotypes in the cohort (Fig. S2 A). When controls and vaccinees were stratified according to their virological status (viremics, controllers, aviremics), there was no direct evidence of MHC effects (Fig. S2 B). The macaque AH776 carrying the MHC IA M3, IB M3 and MHC class II M1 and M3 haplotypes was aviremic. However, this pattern did not discriminate this macaque from other macaques bearing almost identical haplotypes (Table S3). The MHC IB haplotype impacted on the viremia in two out of nine control macaques during the acute phase of the infection (p = 0.047), while no effects were evident among vaccinees (p = 0.086) (Table S4). Similarly, there was no apparent association of MHC with anti-Tat humoral responses (Table S5).

## Discussion

We reported here the safety, immunogenicity and efficacy of a regimen of immunization in cynomolgus macaques based on priming with the HIV-1 native Tat protein adsorbed onto the surface of novel anionic polymeric microspheres (H1D) followed by boosting with Tat and Alum.

The humoral responses generated by the Tat/H1D vaccine formulation differed from those elicited by systemic Tat protein (10 µg) administered with conventional adjuvants (RIBI, Alum), in the absence of H1D microspheres [26]. In fact, in these previous studies the anti-Tat antibody responses became detectable after the second Tat/Alum administration, whereas in the Tat/H1D/Alum protocol of immunization both IgM and IgG anti-Tat responses became clearly detectable only after the first Tat/Alum boost in most vaccinated monkeys, mimicking the kinetics of responses observed following DNA immunization [36]. The lack of detectable antibodies upon priming with Tat/H1D was unexpected and it remains to be clarified whether it depends on the nature of the antigen formulation, the dose, route, or schedule of administration [39], [40], [41].

Nevertheless the kinetics and type of response to the Tat/Alum boosters clearly indicates that the administration of the Tat/H1D complex had primed the macaque’s immune system, generating a large pool of memory B and T cells and driving a balanced Th1- and Th2-type immune responses despite the use of Alum, a known Th2 adjuvant. In fact, both antibody and T-cell (IFN-γ, IL-2 and IL-4) responses were boosted by the first Tat/Alum administration and the majority of the vaccinees maintained these balanced Th1/Th2 responses until challenge, with the exception of IFN-γ, which declined over time. Although limited to IFN-γ, such mixed responses had been already observed in cynomolgus monkeys vaccinated with Tat/Alum [42] and in mice, where Tat-coated anionic nanoparticles induced a Th1 biased immune responses with anti-Tat antibody response levels similar to those observed in mice immunized with Tat/Alum [43]. Similarly, we had previously shown that intradermic administration of Tat in macaques induces Th1-type polarized adaptive immune responses [44]. Likely, these differences are dependent on the animal species and on regimen and schedule of antigen administration.

Upon virus challenge, the Tat/H1D/Alum regimen of immunization resulted in a clear clinical benefit. In fact, vaccinees had a peak viremia significantly lower than controls and also exhibited significant lower depletion (acute phase) or higher recovery (post-acute and chronic phases) rates of CD4^+^ T cells as compared to controls. In particular, 4 vaccinees maintained pre-challenge levels of CD4^+^ T cells throughout the study.

Infection did not elicit humoral or T-cell mediated anamnestic responses to Tat, and pre-challenge cellular responses apparently did not correlate with the virological outcome. Conversely and of interest, we observed that vaccinees with persistent anti-Tat antibodies had a significant advantage in the control of viral replication (controllers) and CD4+ T cell depletion, and a better clinical outcome than viremic vaccinees (viremics) with low or undetectable anti-Tat responses. This is consistent with a number of studies in which anti-Tat antibody persistence was observed in infected individuals resulting in a clinical benefit. In fact, cross-sectional and longitudinal studies in HIV-1 infected patients have shown that anti-Tat antibodies correlate with a slower progression to AIDS [45], [46], [47], [48], [49], [50], [51]. Similarly, anti-Tat antibodies induced by vaccination have been reported to block the activity of extracellular Tat on cell entry, gene expression and replication [31], [52], [53], [54], [55].

Yet the mechanism(s) underlying both the low frequency of anti-Tat antibodies in HIV-1 infected individuals as well as their persistence in the few that mount an anti-Tat antibody response are unknown. Nevertheless, it appears that the very same occurs in macaques infected with a SHIV carrying the HIV-1 *tat* gene. In fact, SHIV89.6P-infected control macaques did not mount detectable anti-Tat humoral responses, whereas vaccinees did not show anamnestic responses to Tat, upon infection. These are consistent findings, since we never detected anti-Tat antibodies in the plasma of SHIV 89.6P-infected cynos also in our previous trials in macaques [26], [29], [35]. The Tat protein is immunogenic as it induces both T-cell and humoral responses in animal models when used as a vaccine, even without adjuvant [25], [26], [29], [44], [56]. Furthermore, Tat protein is released from infected cells [33] and therefore the generation of anti-Tat antibodies would be expected. Yet, only a small fraction (from 10 to 20%) of HIV-1 infected individuals exhibit detectable serum levels of anti-Tat antibodies [46], [57], [58], [59], whereas antibody responses to virtually all other viral proteins are readily induced and detected [60]. Strikingly, this does not occur in HIV-2 infection [61], or, in the SIV macaque model, in that monkeys vaccinated with SIV-Tat exhibit anamnestic responses upon challenge with SIVmac251 [5], [62]. The mechanism(s) underlying this phenomenon is unknown. It is tempting to speculate that in the context of HIV-1 infection Tat conceals itself, an ability that may relate to the highly unstructured and very flexible conformation of the protein [63], as well as to its known ability to bind through the basic region the heparan sulfate proteoglycans present in the extracellular matrix and on the cell membrane [64], thus avoiding antibody recognition and priming (or boosting) of B cell responses. Further studies are needed to address this finding.

More recently, using a novel biopanning strategy, which allows a complete and unbiased profiling of the antibody repertoire and identification of epitopes associated with vaccine protection, antibodies against the amino-terminus (NT) of Tat were reported to be present only in completely or partially protected vaccinated rhesus macaques and to neutralize the trans-activating activity of Tat [65]. In particular, a mimotope was identified (LEPWK) to which Tat-neutralising antibodies present in plasma of protected macaques were directed. Of importance Tat-neutralising antibodies inversely correlated with peak viremia [65]. In our study, all vaccinees responded to peptide aa 1–15 containing the LEPWK mimotope but, even though the 4 controllers exhibited more frequent and higher responses than viremics, the differences did not reach statistically significance. With respect to clinical progression of the infection, responses directed to the aa 46–70 region (mainly core and basic regions of Tat) seem to be dispensable for protection or to have a negative prognostic values. In fact, viremics exhibited a higher, although not significant, response within this region with respect to controllers. In contrast, the protective status among the 4 controllers was associated with responses to GSQTHQVSLSKQPTS and QSRGDPTGPK epitopes present within peptide aa 61–75 and peptide aa 71–85, respectively. These two peptides encompass the glutamine-rich and the integrin-binding RGD domain of Tat, respectively. We have recently described a mechanism that supports these data [31]. We reported that the cystein-rich region of Tat engages the Env V3 loop whereas the RGD motif remains exposed and binds to RGD-binding integrin receptors present on dendritic cells. As a result, Tat re-directs oligomeric Env to the RGD-binding integrin endocytic pathway via a mechanism that is entirely independent of Tat-mediated activation of HIV transcription, thus representing an alternative pathway of virus entry and infection for dendritic cells, monocyte-macrophages, activated endothelial cells [31]. Of importance, this novel entry pathway is insensitive to neutralization by anti-Env antibodies, for which anti-Tat antibodies are needed [31], thus providing a mechanism for the role of anti-Tat antibodies in the control of viral replication. In this regard, it is worth to underscore that Tat is released by acutely infected T cells in the extracellular milieu, enters neighbor cells and exert multiple effects on bystander cells, either directly or indirectly, which result in increased immune cell recruitment and activation and increased viral infection and transmission. Thus, antibodies recognizing these functional domains of Tat may impair Tat’s biological activities [66], decrease viral replication and pathogenesis and therefore contribute to maintaining the non-progression status. The relevance of these responses have been, although indirectly, demonstrated in a trial where the lack of protection was attributable to the failure to elicit antibodies able to block HIV entry via integrin-mediated pathway [67]. According to the authors, the loss of biological activity, conceivably due to oxidation, of the Tat protein used as immunogen, might have been responsible for the lack of induction of protective immunity [67]. These observations further demonstrate the importance of the immunization regimen and of a delivery system able to maintain the fully biological activity of Tat.

In humans, IgG1 antibodies are associated with Th1 and Th2 responses, IgG2 and IgG4 with Th1 and Th2 responses, respectively, while IgG3 are linked to the Th1 pattern. The nature and dose of the antigen, the type of adjuvant used in the vaccine formulation, as well as the system of vaccine delivery, may all influence the IgG subclasses profile [68], [69], [70], [71]. Thus, vaccinees receiving HIV-1 gp120 in Alum exhibited higher Th-2 associated IgG4 responses [72], whereas individuals primed with canarypox-vectored multigenic vaccine showed a prevalence of IgG1 responses [73].

Here we have shown that anti-Tat IgG3 and IgG4 significantly correlate with Tat-specific proliferative responses rather than IFN-γ, IL-2, or IL-4 cytokine production. This association is suggestive of a noncasual link between the Tat protein and T cell helper functions likely driving B cells activities. In fact, besides its role in the virus life cycle and among the other immune-dysregulatory activities, Tat has been reported to induce IL-10 [74], [75], which may modulate the production of IgG4 [76] or induce B cells to secrete IgG1 and IgG3 antibodies [77].

Notably, a significant impact of IgG1, and to a lesser extent IgG3 and IgG4, anti-Tat antibody titers on control of viremia in the acute and post-acute phases of the infection was apparent. IgG1 and IgG3 are the most functional of the subclasses in that they have been associated with HIV-1 neutralization, complement fixation, FcR binding and ADCC/ADCVI [79,79]. Our in vivo results are consistent with other data indicating that interaction of Fc receptor-bearing effector cells with antibody-complexed infected cells is important in reducing virus yield from infected cells and, according to a trial in macaques, to represent a potential correlate of protection [80]. Interestingly, IgG3 anti-gp120 antibodies were detected more frequently in the non-progressor than in the progressor group [73] and, in a case-control study in the RV144 trial, higher V1/V2 specific anti-Env IgG3 responses correlated with a decreased risk of infection acquisition [81]. Further, change from IgG1 to IgG3 of the human monoclonal antibody F105 (IgG1k), which binds the CD4-binding site of gp120, results in a neutralization of T-cell line adapted isolate that was resistant to neutralization by parental IgG1 antibody [82], [83]. Additional studies are ongoing to verify whether IgG1, IgG3 or IgG4 antigen specific profile could be predictive of protective responses in vaccine trials.

To investigate the contribution of host responses to the susceptibility to infection or to the control of viral replication, the MHC class I and II haplotypes of all monkeys were determined. In a previous work we reported that the MHC class IB M1 haplotype correlated with a high viral load (P = 0.0280) and CD4 loss (P = 0.0343), whereas the MHC class IB M2 and M6 haplotypes correlated with increased susceptibility (P = 0.0199) and resistance (P = 0.0087) to infection, respectively [38]. In the present study the association of the MHC class IB M1 haplotype with a worse outcome of the infection was apparent in the 2 control macaques carrying this haplotype, but not in the 4 class IB M1 positive vaccinated monkeys, suggesting that the Tat vaccine effect had successfully counteracted the detrimental impact of class IB M1 haplotype on the course of infection. However, we were unable to confirm the influence of the class IB M2 and M6 haplotypes on the outcome/course of the infection due to the insufficient numerosity.

In a more recent work we reported that the M4 haplotype impacts on proviral copy number and CD4+T cell counts [84]. In particular, lower proviral DNA loads in MHC class IA M4 macaques and lower CD4+T cell counts in MHC class IA, IB, and class II M4 animals were detected during the acute and chronic phases of infection, respectively [84]. In control macaques lower levels of anti-Env binding antibodies were associated with the MHC class IB M4 haplotype in the post acute phase of the infection, whereas lower levels of neutralizing antibodies were found in MHC class IA and IB M4 animals [85] **.** We speculated that during the early phase of infection M4 animals might have been poorly able to control infection as a consequence of a limited antibody response. However in our setting, the impact of MHC haplotypes on viremia, CD4+T cells counts and anti-Tat humoral responses was hardly to define likely due to the limited size of the groups and the inter-individual variability of MHC haplotypes.

A number of preventative vaccine approaches differing in composition of vaccine antigens, route of administration, delivery systems, adjuvants and challenge viruses (SIV/SHIV) have been studied for efficacy in preclinical models [1], [4], [26], [62], [86], resulting in various levels of protection. Although our vaccine regimen did not prevent infection, it did result in a significant control of viral replication and disease progression. Of note, in a study in which rhesus macaques were vaccinated with nanoparticles carrying HIV-gp120, enhancement of infection was observed despite the strong immune responses to gp120 induced, underscoring the relevance of the present findings [87].

Overall, the results of our study indicate that the Tat/H1D/Alum regimen represents an efficient system for vaccination with the native HIV-1 Tat protein or other subunit vaccines for which maintenance of native conformation and biological activities are absolutely required. It seems also evident that the Tat/H1D/Alum regimen of immunization represents a useful way to elicit balanced T cell and humoral protective responses and hold promise for next generation clinical trials.

Finally, our findings provide novel information on anti-Tat responses that may be of relevance in HIV pathogenesis and design of new vaccine antigens.

## Supporting Information

Figure S1 **T cell immune responses to Tat after challenge.** The proliferative responses of (**A**) control and (**E**) vaccinated monkeys are indicated as stimulation index (S.I). In the other left panels are reported the frequencies of SFC/10^6^ cells for (**B**) IFN-γ, (**C**) IL-2 and (**D**) IL-4, respectively, in control monkeys. In the corresponding panels on the right are reported the frequencies of SFC/10^6^ cells for (**F**) IFN-γ, (**G**) IL-2 and (**H**) IL-4 in vaccinees. The dashed line represents the cut-off (SI or SFC/10^6^ PBMCs) of the assay. Samples yielding for IFN-γ ≥80 SFC/10^6^ cells and a fold increase ≥2.5, for IL-2≥30 SFC/10^6^ cells and a fold increase ≥3, and for IL-4≥20 SFC/10^6^ cells and a fold increase ≥2.5 were considered positive as described in the Material and Methods.
(TIF)

Figure S2 **Frequency of MHC class IA, class IB and class II and recombinant haplotypes in the animals included in the study.** **A**) Percentage of animals carrying specific MHC class I haplotypes. MHC class IA: M1 = 44, M2 = 28, M3 = 56, M4 = 22, M5 = 11, M6 = 17; frequency of MHC class IB: M1 = 33, M2 = 28, M3 = 56, M4 = 11, M5 = 11, M6 = 11, recombinant haplotypes = 3; frequency of MHC class II: M1 = 50, M2 = 22, M3 = 56, M4 = 22, M5 = 22, M6 = 17, M7 = 3. **B**) Aviremic, Controller and Viremic macaques stratified by MHC class of haplotypes, The number in parenthesis below the x axis indicates the number (N) of monkeys carrying the indicated haplotype.
(TIF)

Table S1 **Comparison of differences of physical and hematological parameters (4–50 weeks) in naive, H1D-treated and Tat/H1D-vaccinated cynomolgus monkeys.**
(DOCX)

Table S2 **Correlation (nonparametric Spearman rank analyses) between anti-Tat IgG titers and the subclass profile in Tat-vaccinated macaques determined at week 44 before challenge.**
(DOCX)

Table S3 **Summary of data reporting MHC haplotypes, viral load, CD4+ T cell counts and anti-Tat IgG antibodies in samples of control and vaccinated macaques during the acute and chronic phase of the infection.**
(DOCX)

Table S4 **Impact of the MHC haplotype on acute viremia and disease progression in control and vaccinated macaques during the acute (2–4 weeks), post acute (8–16 weeks) and chronic (22–74) phase of the infection with SHIV89.6P.**
(DOCX)

Table S5 **Impact of MHC IB haplotype on antibody responses in vaccinated monkeys as observed during the acute (2–4 weeks), post acute (8–16 weeks) and chronic (22–74) phase of the infection with SHIV89.6p.**
(DOCX)

## References

[1] Sernicola L, Corrias F, Koanga-Mogtomo ML, Baroncelli S, Di Fabio S, et al (1999) Long-lasting protection by live attenuated simian immunodeficiency virus in cynomolgus monkeys: no detection of reactivation after stimulation with a recall antigen. Virology 256: 291–302. 10191194 10.1006/viro.1999.9652

[2] Goletti D, Macchia I, Leone P, Pace M, Sernicola L, et al (2006) Innate anti-viral immunity is associated with the protection elicited by the simian immunodeficiency virus (SIV) live attenuated virus vaccine in cynomolgus monkeys. Med Sci Monit 12: BR330–BR340. 17006396

[3] Manoussaka MS, Berry N, Ferguson D, Stebbings R, Robinson M, et al (2013) Conditionally-live attenuated SIV upregulates global T effector memory cell frequency under replication permissive conditions. Retrovirology 10: 59. 23738926 10.1186/1742-4690-10-59 PMC3706341

[4] Hansen SG, Piatak M Jr, Ventura AB, Hughes CM, Gilbride RM, et al (2013) Immune clearance of highly pathogenic SIV infection. Nature 502: 100–104. 24025770 10.1038/nature12519 PMC3849456

[5] Negri DR, Baroncelli S, Michelini Z, Macchia I, Belli R, et al (2001) Effect of vaccination with recombinant modified vaccinia virus Ankara expressing structural and regulatory genes of SIV(macJ5) on the kinetics of SIV replication in cynomolgus monkeys. J Med Primatol 30: 197–206. 11555138 10.1034/j.1600-0684.2001.d01-53.x

[6] Falivene J, Del Medico Zajac MP, Pascutti MF, Rodrıguez AM, Maeto C, et al (2012) Improving the MVA Vaccine Potential by Deleting the Viral Gene Coding for the IL-18 Binding Protein. PLoS ONE 7: e32220. 22384183 10.1371/journal.pone.0032220 PMC3285208

[7] Allaoui-Attarki K, Fattal E, Pecquet S, Trolle S, Chachaty E, et al (1998) Mucosal immunogenicity elicited in mice by oral vaccination with phosphorylcholine encapsulated in poly (D,L-lactide-co-glycolide) microspheres. Vaccine 16: 685–691. 9562687 10.1016/s0264-410x(97)00261-2

[8] Manocha M, Pal PC, Chitralekha KT, Thomas BE, Tripathi V, et al (2005) Enhanced mucosal and systemic immune response with intranasal immunization of mice with HIV peptides entrapped in PLG microparticles in combination with Ulex Europaeus-I lectin as M cell target. Vaccine 23: 5599–5617. 16099080 10.1016/j.vaccine.2005.06.031

[9] Tamber H, Johansen P, Merkle HP, Gander B (2005) Formulation aspects of biodegradable polymeric microspheres for antigen delivery. Adv Drug Deliv Rev 57: 357–376. 15560946 10.1016/j.addr.2004.09.002

[10] O'Hagan DT, Singh M, Ulmer JB (2006) Microparticle-based technologies for vaccines. Methods 40: 10–19. 16997709 10.1016/j.ymeth.2006.05.017

[11] Nayak B, Panda AK, Ray P, Ray AR (2009) Formulation, characterization and evaluation of rotavirus encapsulated PLA and PLGA particles for oral vaccination. J Microencapsul 26: 154–165. 18608800 10.1080/02652040802211709

[12] Walter E, Moelling K, Pavlovic J, Merkle HP (1999) Microencapsulation of DNA using poly(DL-lactide-co-glycolide): stability issues and release characteristics. J Control Release 61: 361–374. 10477808 10.1016/s0168-3659(99)00151-0

[13] van de Weert M, Hennink WE, Jiskoot W (2000) Protein instability in poly(lactic-co-glycolic acid) microparticles. Pharm Res 17: 1159–1167. 11145219 10.1023/a:1026498209874

[14] Singh M, Chesko J, Kazzaz J, Ugozzoli M, Kan E, et al (2004) Adsorption of a novel recombinant glycoprotein from HIV (Env gp120dV2 SF162) to anionic PLG microparticles retains the structural integrity of the protein, whereas encapsulation in PLG microparticles does not. Pharm Res 21: 2148–2152. 15648244 10.1007/s11095-004-7666-6

[15] Laus M, Sparnacci K, Ensoli B, Buttò S, Caputo A, et al (2001) Complex associates of plasmid DNA and a novel class of block copolymers with PEG and cationic segments as new vectors for gene delivery. J Biomater Sci Polym 12: 209–228. 10.1163/156856201750180933 11403237

[16] Chesko J, Kazzaz J, Ugozzoli M, O'Hagan DT, Singh M (2005) An investigation of the factors controlling the adsorption of protein antigens to anionic PLG microparticles. J Pharm Sci 94: 2510–2519. 16200615 10.1002/jps.20472

[17] Singh M, Kazzaz J, Ugozzoli M, Malyala P, Chesko J, et al (2006) Polylactide-co-glycolide microparticles with surface adsorbed antigens as vaccine delivery systems. Curr Drug Deliv 3: 115–120. 16472100 10.2174/156720106775197565

[18] Taman-Onal Y, Munier S, Ganee A, Terrat C, Durand PY, et al (2006) Surfactant-free anionic PLA nanoparticles coated with HIV-1 p24 protein induced enhanced cellular and humoral immune responses in various animal models. J Control Release 112: 175–185. 16563545 10.1016/j.jconrel.2006.02.006

[19] Lamalle-Bernard D, Munier S, Compagnon C, Charles MH, Kalyanaraman VS, et al (2006) Coadsorption of HIV-1 p24 and gp120 proteins to surfactant-free anionic PLA nanoparticles preserves antigenicity and immunogenicity. J Control Release 115: 57–67. 16919350 10.1016/j.jconrel.2006.07.006

[20] Caputo A, Sparnacci K, Ensoli B, Tondelli L (2008) Functional polymeric nano/microparticles for surface adsorption and delivery of protein and DNA vaccines. Curr Drug Deliv 5: 230–242. 18855591 10.2174/156720108785914961

[21] Voltan R, Castaldello A, Brocca-Cofano E, De Michele R, Triulzi C, et al (2009) Priming with a very low dose of DNA complexed with cationic block copolymers followed by protein boost elicits broad and long-lasting antigen-specific humoral and cellular responses in mice.Vaccine. 27: 4498–44507. 10.1016/j.vaccine.2009.05.031 19450649

[22] Sparnacci K, Laus M, Tondelli L, Bernardi C, Magnani L, et al (2005) Core-shell microspheres by dispersion polymerization as promising delivery systems for proteins. J Biomater Sci Polym Ed 16: 1557–1574. 16366337 10.1163/156856205774576673

[23] Caputo A, Brocca-Cofano E, Castaldello A, De Michele R, Altavilla G, et al (2004) Novel biocompatible anionic polymeric microspheres for the delivery of the HIV-1 Tat protein for vaccine application. Vaccine 22: 2910–2924. 15246628 10.1016/j.vaccine.2003.12.025

[24] Voltan R, Castaldello A, Brocca-Cofano E, Altavilla G, Caputo A, et al (2007) Preparation and characterization of innovative protein-coated poly(methylmethacrylate) core-shell nanoparticles for vaccine purposes. Pharm Res 24: 1870–1882. 17476465 10.1007/s11095-007-9310-8

[25] Caputo A, Castaldello A, Brocca-Cofano E, Voltan R, Bortolazzi F, et al. 2009 Induction of humoral and enhanced cellular immune responses by novel core-shell nanosphere- and microsphere-based vaccine formulations following systemic and mucosal administration. Vaccine 27: 3605–3615. 19464541 10.1016/j.vaccine.2009.03.047

[26] Cafaro A, Caputo A, Fracasso C, Maggiorella MT, Goletti D, et al (1999) Control of SHIV-89.6P-infection of cynomolgus monkeys by HIV-1 Tat protein vaccine. Nat Med 5: 643–650. 10371502 10.1038/9488

[27] Ensoli B, Fiorelli V, Ensoli F, Lazzarin A, Visintini R, et al (2008) The therapeutic phase I trial of the recombinant native HIV-1 Tat protein. AIDS 22: 2207–2209. 18832884 10.1097/QAD.0b013e32831392d4

[28] Longo O, Tripiciano A, Fiorelli V, Bellino S, Scoglio A, et al (2009) Phase I therapeutic trial of the HIV-1 Tat protein and long term follow-up. Vaccine 27: 3306–3312. 19208456 10.1016/j.vaccine.2009.01.090

[29] Maggiorella MT, Baroncelli S, Michelini Z, Fanales-Belasio E, Moretti S, et al (2004) Long-term protection against SHIV89.6P replication in HIV-1 Tat vaccinated cynomolgus monkeys. Vaccine 22: 3258–3269. 15308348 10.1016/j.vaccine.2004.03.009

[30] Ensoli B, Bellino S, Tripiciano A, Longo O, Francavilla V, et al (2010) Therapeutic immunization with HIV-1 Tat reduces immune activation and loss of regulatory T-cells and improves immune function in subjects on HAART. PLoS ONE 5: e13540. 21085635 10.1371/journal.pone.0013540 PMC2978690

[31] Monini P, Cafaro A, Srivastava IK, Moretti S, Sharma VA, et al (2012) HIV-1 Tat Promotes Integrin-Mediated HIV Transmission to Dendritic Cells by Binding Env Spikes and Competes Neutralization by Anti-HIV Antibodies. PLoS ONE 7: e48781. 23152803 10.1371/journal.pone.0048781 PMC3496724

[32] Barillari G, Sgadari C, Fiorelli V, Samaniego F, Colombini S, et al (1999) The Tat protein of human immunodeficiency virus type-1 promotes vascular cell growth and locomotion by engaging the alpha5beta1 and alphavbeta3 integrins and by mobilizing sequestered basic fibroblast growth factor. Blood 94: 663–672. 10397733

[33] Ensoli B, Buonaguro L, Barillari G, Fiorelli V, Gendelman R, et al (1993) Release, uptake, and effects of extracellular human immunodeficiency virus type 1 Tat protein on cell growth and viral transactivation. J Virol 67: 277–287. 8416373 10.1128/jvi.67.1.277-287.1993 PMC237361

[34] Borsetti A, Baroncelli S, Maggiorella MT, Bellino S, Moretti S, et al (2008) Viral outcome of simian-human immunodeficiency virus SHIV-89.6P adapted to cynomolgus monkeys. Arch Virol 153: 463–472. 18080858 10.1007/s00705-007-0009-2

[35] Ferrantelli F, Maggiorella MT, Schiavoni I, Sernicola L, Olivieri E, et al (2011) A combination HIV vaccine based on Tat and Env proteins was immunogenic and protected macaques from mucosal SHIV challenge in a pilot study. Vaccine 29: 2918–2932. 21338681 10.1016/j.vaccine.2011.02.006

[36] Cafaro A, Titti F, Fracasso C, Maggiorella MT, Baroncelli S, et al (2001) Vaccination with DNA containing tat coding sequences and unmethylated CpG motifs protects cynomolgus monkeys upon infection with simian/human immunodeficiency virus (SHIV89.6P). Vaccin*e* 19: 2862–2877. 11282197 10.1016/s0264-410x(01)00002-0

[37] Wiseman RW, Wojcechowskyj JA, Greene JM, Blasky AJ, Gopon T, et al (2007) Simian immunodeficiency virus SIVmac239 infection of major histocompatibility complex-identical cynomolgus macaques from Mauritius. J Virol 81: 349–361. 17035320 10.1128/JVI.01841-06 PMC1797269

[38] Cafaro A, Bellino S, Titti F, Maggiorella MT, Sernicola L, et al (2010) Impact of viral dose and major histocompatibility complex class IB haplotype on viral outcome in mauritian cynomolgus monkeys vaccinated with Tat upon challenge with simian/human immunodeficiency virus SHIV89.6P. J Virol 84: 8953–8958. 10.1128/JVI.00377-10 PMC2919029 20554774

[39] Turner GS (1978) Immunoglobulin (IgG) and (IgM) antibody responses to rabies vaccine. J Gen Virol 40: 595–604. 690611 10.1099/0022-1317-40-3-595

[40] Otten GR, Schaefer M, Doe B, Liu H, Srivastava I, et al (2005) Enhanced potency of plasmid DNA microparticle human immunodeficiency virus vaccines in rhesus macaques by using a priming-boosting regimen with recombinant proteins. J Virol 79: 8189–8200. 15956564 10.1128/JVI.79.13.8189-8200.2005 PMC1143738

[41] Rappuoli R (2007) Bridging the knowledge gaps in vaccine design. Nat Biotechnol 25: 1361–1366. 18066025 10.1038/nbt1207-1361

[42] Turbant S, Martinon F, Moine G, Le Grand R, Léonetti M (2009) Cynomolgus macaques immunized with two HIV-1 Tat stabilized proteins raise strong and long-lasting immune responses with a pattern of Th1/Th2 response differing from that in mice. Vaccine 27: 5349–5356. 19607953 10.1016/j.vaccine.2009.06.083

[43] Cui Z, Patel J, Tuzova M, Ray P, Phillips R, et al (2004) Strong T cell type-1 immune responses to HIV-1 Tat (1–72) protein-coated nanoparticles. Vaccine 22: 2631–2640. 15193389 10.1016/j.vaccine.2003.12.013

[44] Fanales-Belasio E, Moretti S, Fiorelli V, Tripiciano A, Pavone Cossut MR, et al (2009) HIV-1 Tat Addresses Dendritic Cells to Induce a Predominant Th1-Type Adaptive Immune Response That Appears Prevalent in the Asymptomatic Stage of Infection. J Immunol 182: 2888–2897. 19234184 10.4049/jimmunol.0711406

[45] Mediouni S, Darque A, Baillat G, Ravaux I, Dhiver C, et al (2012) Antiretroviral therapy does not block the secretion of the human immunodeficiency virus tat protein. Infect Disord Drug Targets 12: 81–6. 22280310 10.2174/187152612798994939

[46] Bellino S, Tripiciano A, Picconi O, Francavilla V, Longo O, et al (2014) The presence of anti-Tat antibodies in HIV-infected individuals is associated with containment of CD4+ T-cell decay and viral load, and with delay of disease progression: results of a 3-year cohort study. Retrovirology 11: 49. 24961156 10.1186/1742-4690-11-49 PMC4087126

[47] Reiss P, Lange JM, de Ronde A, de Wolf F, Dekker J, et al (1990) Speed of progression to AIDS and degree of antibody response to accessory gene products of HIV-1. J Med Virol 30: 163–168. 2341832 10.1002/jmv.1890300303

[48] Zagury JF, Sill A, Blattner W, Lachgar A, Le Buanec H, et al (1998) Antibodies to the HIV-1 Tat protein correlated with nonprogression to AIDS: a rationale for the use of Tat toxoid as an HIV-1 vaccine. J Hum Virol 1: 282–292. 10195253

[49] Re MC, Vignoli M, Furlini G, Gibellini D, Colangeli V, et al (2001) Antibodies against full-length Tat protein and some low-molecular-weight Tat-peptides correlate with low or undetectable viral load in HIV-1 seropositive patients. J Clin Virol 21: 81–89. 11255101 10.1016/s1386-6532(00)00189-x

[50] Richardson MW, Mirchandani J, Duong J, Grimaldo S, Kocieda V, et al (2003) Antibodies to Tat and Vpr in the GRIV cohort: differential association with maintenance of long-term non-progression status in HIV-1 infection. Biomed Pharmacother 57: 4–14. 12642031 10.1016/s0753-3322(02)00327-x

[51] Rezza G, Fiorelli V, Dorrucci M, Ciccozzi M, Tripiciano A, et al (2005) The presence of anti-Tat antibodies is predictive of long-term nonprogression to AIDS or severe immunodeficiency: findings in a cohort of HIV-1 seroconverters. J Infect Dis 191: 1321–1324. 15776379 10.1086/428909

[52] Belliard G, Hurtrel B, Moreau E, Lafont BA, Monceaux V, et al (2005) Tat-neutralizing versus Tat-protecting antibodies in rhesus macaques vaccinated with Tat peptides. Vaccine. 23: 1399–1407. 10.1016/j.vaccine.2004.08.037 15661389

[53] Devadas K, Boykins RA, Hewlett IK, Wood OL, Clouse KA, et al (2007) Antibodies against a multiple-peptide conjugate comprising chemically modified human immunodeficiency virus type-1 functional Tat peptides inhibit infection. Peptides 28: 496–504. 17188401 10.1016/j.peptides.2006.11.007

[54] Partidos CD, Hoebeke J, Moreau E, Chaloin O, Tunis M, et al (2005) The binding affinity of double-stranded RNA motifs to HIV-1 Tat protein affects transactivation and the neutralizing capacity of anti-Tat antibodies elicited after intranasal immunization. Eur J Immunol 35: 1521–1529. 15789358 10.1002/eji.200425676

[55] Kashi VP, Jacob RA, Paul S, Nayak K, Satish B, et al (2009) HIV-1 Tat-specific IgG antibodies in high-responders target a B-cell epitope in the cysteine-rich domain and block extracellular Tat efficiently. Vaccine 27: 6739–6747. 19744585 10.1016/j.vaccine.2009.08.078

[56] Moreau E, Belliard G, Partidos CD, Pradezinsky F, Le Buanec H, et al (2004) Important B-cell epitopes for neutralization of human immunodeficiency virus type 1 Tat in serum samples of humans and different animal species immunized with Tat protein or peptides. J Gen Virol 85: 2893–2901. 15448351 10.1099/vir.0.80365-0

[57] Butto S, Fiorelli V, Tripiciano A, Ruiz-Alvarez MJ, Scoglio A, et al (2003) Sequence conservation and antibody cross-recognition of clade B human immunodeficiency virus (HIV) type 1 Tat protein in HIV-1-infected Italians, Ugandans, and South Africans. J Infect Dis 188: 1171–1180. 14551888 10.1086/378412

[58] Demirhan I, Chandra A, Mueller F, Mueller H, Biberfeld P, et al (2000) Antibody spectrum against the viral transactivator protein in patients with human immunodeficiency virus type 1 infection and Kaposi's sarcoma. J Hum Virol 3: 137–43. 10881993

[59] Chen Q, Li L, Liao W, Zhang H, Wang J, et al (2013) Characterization of Tat antibody responses in Chinese individuals infected with HIV-1. PLoS One 8: e60825. 23565278 10.1371/journal.pone.0060825 PMC3614898

[60] Binley JM, Klasse PJ, Cao Y, Jones I, Markowitz M, et al (1997) Differential regulation of the antibody responses to Gag and Env proteins of human immunodeficiency virus type 1. J Virol 71: 2799–809. 9060635 10.1128/jvi.71.4.2799-2809.1997 PMC191404

[61] Rodriguez SK, Sarr AD, Olorunnipa O, Popper SJ, Gueye-Ndiaye A, et al (2006) The absence of anti-Tat antibodies is associated with risk of disease progression in HIV-2 infection. J Infect Dis 194: 760–763. 16941341 10.1086/507042

[62] Negri DR, Baroncelli S, Catone S, Comini A, Michelini Z, et al (2004) Protective efficacy of a multicomponent vector vaccine in cynomolgus monkeys after intrarectal simian immunodeficiency virus challenge. J Gen Virol 85: 1191–201. 15105535 10.1099/vir.0.79794-0

[63] Mediouni S, Baillat G, Darque A, Ravaux I, Loret E (2011) HIV-1 infected patients have antibodies recognizing folded Tat. Infect Disord Drug Targets 1: 57–63. 10.2174/187152611794407737 21303342

[64] Chang HC, Samaniego F, Nair BC, Buonaguro L, Ensoli B (1997) HIV-1 Tat protein exits from cells via a leaderless secretory pathway and binds to extracellular matrix-associated heparan sulfate proteoglycans through its basic region. AIDS 11: 1421–1431. 9342064 10.1097/00002030-199712000-00006

[65] Bachler BC, Humbert M, Palikuqi B, Siddappa NB, Lakhashe SK, et al (2013) Novel biopanning strategy to identify epitopes associated with vaccine protection. J Virol 87: 4403–4416. 23388727 10.1128/JVI.02888-12 PMC3624354

[66] Titti F, Cafaro A, Ferrantelli F, Tripiciano A, Moretti S, et al (2007) Problems and emerging approaches in HIV/AIDS vaccine development. Expert Opin Emerg Drugs. 12: 23–48. 10.1517/14728214.12.1.23 17355212

[67] Demberg T, Brocca-Cofano E, Kuate S, Aladi S, Vargas-Inchaustegui DA, et al (2013) Impact of antibody quality and anamnestic response on viremia control post-challenge in a combined Tat/Env vaccine regimen in rhesus macaques.Virology. 440: 210–221. 10.1016/j.virol.2013.02.024 PMC3744165 23528732

[68] Hemmi H, Takeuchi O, Kawai T, Kaisho T, Sato S, et al (2000) Toll-like receptor recognizes bacterial DNA. Nature 408: 740–745. 11130078 10.1038/35047123

[69] Weeratna RD, Brazolot Millan CL, McCluskie MJ, Davis HL (2001) CpG ODN can re-direct the Th bias of established Th2 immune responses in adult and young mice. FEMS Immunol Med Microbiol 32: 65–71. 11750224 10.1111/j.1574-695X.2001.tb00535.x

[70] Visciano ML, Tagliamonte M, Tornesello ML, Buonaguro FM, Buonaguro L (2012) Effects of adjuvants on IgG subclasses elicited by virus-like particles. J Transl Med 10: 4. 22221900 10.1186/1479-5876-10-4 PMC3311067

[71] Lefeber DJ, Benaissa-Trouw B, Vliegenthart JF, Kamerling JP, Jansen WT, et al (2003) Th1-directing adjuvants increase the immunogenicity of oligosaccharide-protein conjugate vaccines related to Streptococcus pneumoniae type 3. Infect Immun 71: 6915–6920. 14638780 10.1128/IAI.71.12.6915-6920.2003 PMC308892

[72] Gorse GJ, Corey L, Patel GB, Mandava M, Hsieh RH, et al (1999) HIV-1MN recombinant glycoprotein 160 vaccine-induced cellular and humoral immunity boosted by HIV-1MN recombinant glycoprotein 120 vaccine. National Institute of Allergy and Infectious Diseases AIDS Vaccine Evaluation Group. AIDS Res Hum Retrovir 15: 115–132. 10029244 10.1089/088922299311547

[73] Banerjee K, Klasse PJ, Sanders RW, Pereyra F, Michael E, et al (2010) IgG subclass profiles in infected HIV type 1 controllers and chronic progressors and in uninfected recipients of Env vaccines. AIDS Res Hum Retroviruses 26: 445–58. 20377426 10.1089/aid.2009.0223 PMC2864049

[74] Li JC, Yim HC, Lau AS (2010) Role of HIV-1 Tat in AIDS pathogenesis: its effects on cytokine dysregulation and contributions to the pathogenesis of opportunistic infection. AIDS 24: 1609–1623. 20588103 10.1097/QAD.0b013e32833ac6a0

[75] Badou A, Bennasser Y, Moreau M, Leclerc C, Benkirane M, et al (2000) Tat protein of human immunodeficiency virus type 1 induces interleukin-10 in human peripheral blood monocytes: implication of protein kinase C-dependent pathway. J Virol 74: 10551–10562. 11044099 10.1128/jvi.74.22.10551-10562.2000 PMC110929

[76] Satoguina JS, Weyand E, Larbi J, Hoerauf A (2005) T regulatory-1 cells induce IgG4 production by B cells: role of IL-10. J Immunol 174: 4718–4726. 15814696 10.4049/jimmunol.174.8.4718

[77] Brière F, Servet-Delprat C, Bridon JM, Saint-Remy JM, Banchereau J (1994) Human interleukin 10 induces naive surface immunoglobulin D+ (sIgD+) B cells to secrete IgG1 and IgG3. J Exp Med. 179: 757–762. 10.1084/jem.179.2.757 PMC2191366 8294883

[78] Tomaras GD, Haynes BF (2009) HIV-1-specific antibody responses during acute and chronic HIV-1 infection. Curr Opin HIV AIDS 4: 373–379. 20048700 10.1097/COH.0b013e32832f00c0 PMC3133462

[79] Hessell AJ, Hangartner L, Hunter M, Havenith CEG, Beurskens FJ, et al (2007) Fc receptor but not complement binding is important in antibody protection against HIV. Nature 449: 101–104. 17805298 10.1038/nature06106

[80] Florese RH, Demberg T, Xiao P, Kuller L, Larsen K, et al (2009) Contribution of nonneutralizing vaccine-elicited antibody activities to improved protective efficacy in rhesus macaques immunized with Tat/Env compared with multigenic vaccines. J Immunol 182: 3718–3727. 19265150 10.4049/jimmunol.0803115 PMC2744397

[81] Gottardo R, Bailer RT, Korber BT, Gnanakaran S, Phillips J, et al (2013) Plasma IgG to Linear Epitopes in the V2 and V3 Regions of HIV-1 gp120 Correlate with a Reduced Risk of Infection in the RV144 Vaccine Efficacy Trial. PLoS ONE 8: e75665. 24086607 10.1371/journal.pone.0075665 PMC3784573

[82] Cavacini LA, Ernes C, Power J, Desharnais FD, Duval M, et al (1995) Influence of Heavy Chain Constant Regions on Antigen Binding and HIV-1 Neutralization by a Human Monoclonal Antibody. J Immunol 155: 3638–3644. 7561063

[83] Cavacini LA, Kuhrt D, Duval M, Mayer K, Posner MR (2003) Binding and neutralization activity of human IgG1 and IgG3 from serum of HIV-infected individuals. AIDS Res Hu Retrovir Vol 19: 785–792. 10.1089/088922203769232584 14585209

[84] Borsetti A, Maggiorella MT, Sernicola L, Bellino S, Ferrantelli F, et al (2012) Influence of MHC class I and II haplotypes on the experimental infection of Mauritian cynomolgus macaques with SHIVSF162P4cy. Tissue Antigens 80: 36–45. 22494179 10.1111/j.1399-0039.2012.01875.x

[85] Borsetti A, Ferrantelli F, Maggiorella MT, Sernicola L, Bellino S, et al (2014) Effect of MHC haplotype on immune response upon experimental SHIVSF162P4cy infection of Mauritian cynomolgus macaques. PLoS ONE 9: e93235. 24695530 10.1371/journal.pone.0093235 PMC3973703

[86] Barouch DH, Stephenson KE, Borducchi EN, Smith K, Stanley K, et al (2013) Protective efficacy of a global HIV-1 mosaic vaccine against heterologous SHIV challenges in rhesus monkeys. Cell 155: 531–539. 24243013 10.1016/j.cell.2013.09.061 PMC3846288

[87] Himeno A, Akagi T, Uto T, Wang X, Baba M, et al (2010) Evaluation of the immune response and protective effects of rhesus macaques vaccinated with biodegradable nanoparticles carrying gp120 of human immunodeficiency virus. Vaccine 28: 5377–5385. 20472029 10.1016/j.vaccine.2010.04.110

