# Supplementary figures and images for: Biocompatible Anionic Polymeric Microspheres as Priming Delivery System for Effetive HIV/AIDS Tat-Based Vaccines
Source: PLoS One. 2014 Oct 30;9(10):e111360. doi: 10.1371/journal.pone.0111360 (PMC4214729; doi:10.1371/journal.pone.0111360)

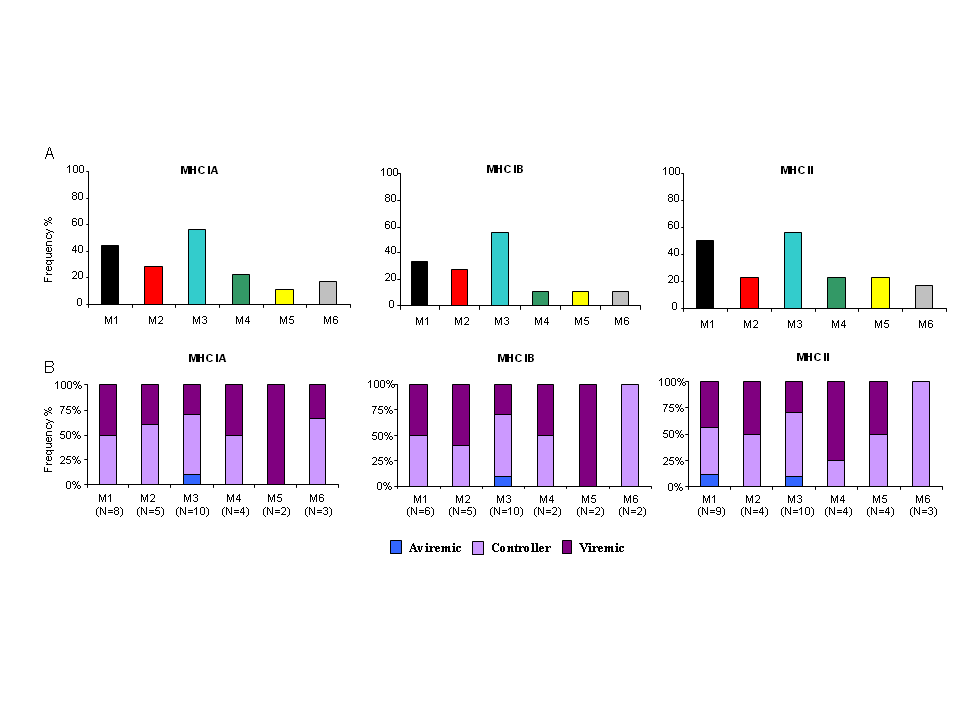

Supplement: Figure S2 — Frequency of MHC class IA, class IB and class II and recombinant haplotypes in the animals included in the study. A) Percentage of animals carrying specific MHC class I haplotypes. MHC class IA: M1 = 44, M2 = 28, M3 = 56, M4 = 22, M5 = 11, M6 = 17; frequency of MHC class IB: M1 = 33, M2 = 28, M3 = 56, M4 = 11, M5 = 11, M6 = 11, recombinant haplotypes = 3; frequency of MHC class II: M1 = 50, M2 = 22, M3 = 56, M4 = 22, M5 = 22, M6 = 17, M7 = 3. B) Aviremic, Controller and Viremic macaques stratified by MHC class of haplotypes, The number in parenthesis below the x axis indicates the number (N) of monkeys carrying the indicated haplotype. (TIF) [file pone.0111360.s002.tif]
