# Supplementary material for: Biocompatible Anionic Polymeric Microspheres as Priming Delivery System for Effetive HIV/AIDS Tat-Based Vaccines
Source: PLoS One. 2014 Oct 30;9(10):e111360. doi: 10.1371/journal.pone.0111360 (PMC4214729; doi:10.1371/journal.pone.0111360)
Supplement: Table S1 — Comparison of differences of physical and hematological parameters (4–50 weeks) in naive, H1D-treated and Tat/H1D-vaccinated cynomolgus monkeys. (DOCX) [file pone.0111360.s003.docx]

**TABLE S1.** **Comparison of physical and hematological parameters (4-50 weeks) in naive, H1D-treated and Tat/H1D-vaccinated cynomolgus monkeys**.

| **Groups** |  | **Weight (gr)** | **WBC** | **RBC** | **PLT** | **HGB** | **MCV** | **MCH** | **MCHC** | **HCT** | **RDW** | **MPV** | **Neutr (%)** | **Eos (%)** | **Bas**  **(%)** | **Lympho (%)** | **Mono (%)** |
| --- | --- | --- | --- | --- | --- | --- | --- | --- | --- | --- | --- | --- | --- | --- | --- | --- | --- |
| **Naive (n= 21)** | **Mean** | 1,102.4 | -523.8 | -48,095.2 | -15,571.4 | -0.1 | 1.3 | 0.0 | -0.6 | 0.5 | 0.0 | 0.3 | 4.2 | -0.1 | 0.0 | -3.7 | -0.3 |
|  | **Std Dev** | 590.3 | 3,606.8 | 409,153.0 | 68,123.8 | 1.3 | 1.4 | 1.3 | 1.7 | 2.5 | 1.0 | 0.8 | 17.5 | 1.7 | 0.2 | 16.7 | 1.2 |
|  | **Min** | 320.0 | -7900.0 | -1,210,000.0 | -157,000.0 | -2.2 | -0.4 | -1.6 | -2.7 | -5.1 | -1.8 | -1.9 | -32.0 | -6.0 | -1.0 | -31.0 | -3.0 |
|  | **Max** | 2,385.0 | 5,400.0 | 480,000.0 | 9,5000.0 | 2.2 | 4.4 | 2.9 | 3.4 | 4.2 | 1.7 | 1.7 | 36.0 | 2.0 | 0.0 | 31.0 | 1.0 |
|  | **t-Test** | <0.0001 | 0.5133 | 0.5961 | 0.3074 | 0.7924 | 0.0003 | 0.8932 | 0.1031 | 0.3649 | 0.9642 | 0.1550 | 0.2866 | 0.8033 | 0.3293 | 0.3190 | 0.2008 |
|  |  |  |  |  |  |  |  |  |  |  |  |  |  |  |  |  |  |
| **Control (n=9)** | **Mean** | 1,123.3 | 1,000.0 | -267,777.8 | 38,777.8 | -0.2 | 1.3 | 0.6 | 0.3 | -0.9 | 0.2 | 0.4 | 7.2 | -0.2 | -0.1 | -7.1 | 0.4 |
|  | **Std Dev** | 909.0 | 4,640.0 | 354,639.9 | 149,151.6 | 0.6 | 1.9 | 0.5 | 0.9 | 1.9 | 1.0 | 0.8 | 9.6 | 0.8 | 0.3 | 9.9 | 0.5 |
|  | **Min** | -210.0 | -5,100.0 | -900,000.0 | -151,000.0 | -1.4 | -1.8 | -0.5 | -0.6 | -3.7 | -1.2 | -0.4 | -5.0 | -2.0 | -1.0 | -28.0 | 0.0 |
|  | **Max** | 2,340.0 | 10,500.0 | 170,000.0 | 388,000.0 | 0.7 | 3.3 | 1.4 | 1.9 | 2.2 | 1.4 | 2.3 | 27.0 | 1.0 | 0.0 | 3.0 | 1.0 |
|  | **t-Test** | 0.0060 | 0.5360 | 0.0533 | 0.4579 | 0.4408 | 0.0687 | 0.0084 | 0.3282 | 0.1809 | 0.4654 | 0.1518 | 0.0532 | 0.4468 | 0.3466 | 0.0642 | 0.0796 |
|  |  |  |  |  |  |  |  |  |  |  |  |  |  |  |  |  |  |
| **Vaccinated (n=9)** | **Mean** | 1,408.3 | 1,455.6 | -7,666.7 | 19,111.1 | 0.4 | 0.4 | 0.9 | 1.2 | -0.3 | 0.5 | 0.2 | 4.8 | -0.3 | 0.1 | -5.2 | 0.7 |
|  | **Std Dev** | 683.1 | 4,894.2 | 23,119.3 | 57,422.7 | 1.3 | 1.5 | 1.6 | 2.5 | 1.8 | 1.3 | 0.6 | 10.4 | 0.7 | 0.3 | 9.9 | 0.7 |
|  | **Min** | 370.0 | -3200.0 | -36,000.0 | -55,000.0 | -1.1 | -1.7 | -0.6 | -1.3 | -3.1 | -1.5 | -0.9 | -9.0 | -2.0 | 0.0 | -20.0 | 0.0 |
|  | **Max** | 2,440.0 | 9,600.0 | 37,000.0 | 129,000.0 | 2.7 | 2.7 | 3.1 | 4.7 | 2.5 | 2.0 | 0.9 | 22.0 | 0.0 | 1.0 | 9.0 | 2.0 |
|  | **t-Test** | 0.0003 | 0.3983 | 0.3489 | 0.3473 | 0.4095 | 0.4906 | 0.1204 | 0.1947 | 0.6386 | 0.2626 | 0.2905 | 0.2055 | 0.1950 | 0.3466 | 0.1521 | 0.0222 |

Student’s t-test was used to evaluate the difference between vaccinated and control monkeys for hematological parameters**.**

A statistically significant increase of the body weight was observed in control (p= 0.0060), vaccinees (p=0.0003) and naïve (p=<0.0001) monkeys. However the weight gain was expected as it occurred over almost 1 year and could not be associated to the specific treatments. Similarly, other variations such as the increase of MCH values (p=0.0084) among controls, the number of monocytes (p=0.022) in vaccinees or MCV values in naïve (p=0.0003) were observed which were likely associated to the age rather than to the treatment [1, 2, 3].

RBC: red blood cell count; Hb: hemoglobin; MCV: mean corpuscular volume; MCH: mean corpuscular hemoglobin; WBC: total white blood cell count; Lympho: lymphocytes; Neut: neutrophils; Eos: eosinophils; Bas:basophils; Mono: monocytes.

**Supplementary References**

1. Baroncelli S, Panzini G, Geraci A, Pardini S, Corrias F, Iale E et al. (1997) Longitudinal characterization of CD4, CD8 T-cell subsets and of haematological parameters in healthy newborns of cynomolgus monkeys Vet Immunol Immunopathol59:141-150.

2. Drevon-Gaillot E, Perron-Lepage MF, Clement C, Burnett R. (2006) A review of background findings in cynomolgus monkeys (Macaca fascicularis) from three different geographical origins. Exp Toxicol Pathol;58:77-88.

3. Mahaney MC, Brugnara C, Lease LR, Platt OS. Genetic influences on peripheral blood cell counts: a study in baboons. (2005) Blood106:1210-1214.
