# Supplementary material for: Biocompatible Anionic Polymeric Microspheres as Priming Delivery System for Effetive HIV/AIDS Tat-Based Vaccines
Source: PLoS One. 2014 Oct 30;9(10):e111360. doi: 10.1371/journal.pone.0111360 (PMC4214729; doi:10.1371/journal.pone.0111360)
Supplement: Table S2 — Correlation (nonparametric Spearman rank analyses) between anti-Tat IgG titers and the subclass profile in Tat-vaccinated macaques determined at week 44 before challenge. (DOCX) [file pone.0111360.s004.docx]

**TABLE S2. Correlation (nonparametric Spearman rank analyses) between anti-Tat IgG titers and the subclass profile in Tat-vaccinated macaques determined at week 44 before challenge**.

|  | IgG | IgG1 | IgG2 | IgG3 |
| --- | --- | --- | --- | --- |
| IgG1 | r = 0.248  p = 0.520 |  |  |  |
| IgG2 | r = 0.4448  p = 0.229 | **r = 0.776**  **p = 0.017** |  |  |
| IgG3 | r = 0.615  p = 0.085 | r = 0.542  p = 0.132 | **r = 0.821**  **p = 0.0083** |  |
| IgG4 | r = 0.626  p = 0.076 | **r = 0.821**  **p = 0.0083** | **r = 0.785**  **p = 0.017** | **r = 0.794**  **p = 0.0138** |
