# Supplementary material for: Biocompatible Anionic Polymeric Microspheres as Priming Delivery System for Effetive HIV/AIDS Tat-Based Vaccines
Source: PLoS One. 2014 Oct 30;9(10):e111360. doi: 10.1371/journal.pone.0111360 (PMC4214729; doi:10.1371/journal.pone.0111360)
Supplement: Table S3 — Summary of data reporting MHC haplotypes, viral load, CD4+ T cell counts and anti-Tat IgG antibodies in samples of control and vaccinated macaques during the acute and chronic phase of the infection. (DOCX) [file pone.0111360.s005.docx]

| Monkeys and Treatment | | | MHC  1A | |  | MHC  1B | |  | MHC  2DP | |  | MHC  2DQ | |  | MHC  2DR | | Plasma Viral RNA  (Eq/ml) | | CD4+ T Cells counts  (cells/mmc) | | Anti-Tat IgG Ab  Titers | |
| --- | --- | --- | --- | --- | --- | --- | --- | --- | --- | --- | --- | --- | --- | --- | --- | --- | --- | --- | --- | --- | --- | --- |
|  | | | 1 | 2 |  | 1 | 2 |  | 1 | 2 |  | 1 | 2 |  | 1 | 2 | Acute  phase^a)^ | Chronic phase^b)^  (range) | Acute phase ^c)^ | Chronic phase (range) | Day of challenge | Chronic phase (range) |
| Controls | Viremics | AC032 | M1 | M5 |  | M1 | M2 |  | M2 | M1 |  | M2 | M4 |  | M2 | M4 | 1.1x10^7^ | 0.9-3x10^3d)^ | 36 (96.4) | 42-47 | - | - |
|  |  | AC601 | M1 | M4 |  | M1 | M3 |  | M1 | M3 |  | M1 | M3 |  | M1 | M3 | 4.3x10^6^ | <50-3x10^3^ | 151 (83.9) | 67-396 | - | - |
|  |  | AC739 | M1 | M3 |  | M3 | M5 |  | M3 | M5 |  | M3 | M5 |  | M3 | M5 | 8.8x10^5^ | 1.3-5.9x10^3d)^ | 56 (97.2) | 336-789 | - | - |
|  |  | AG249 | M3 | M4 |  | M4 |  |  | M4 | M4 |  | M4 | M4 |  | M4 | M4 | 3.3 x10^6^ | 1.3-5x10^3^ | 69 (90.4) | 32-186 | - | - |
|  |  | AI075 | M3 | M6 |  | M3 | M6 |  | M3 | M6 |  | M3 | M6 |  | M3 | M6 | 1.4 x10^6^ | <50-1.3x10^2^ | 47 (97.9) | 450-1,252 | - | - |
|  |  |  |  |  |  |  |  |  |  |  |  |  |  |  |  |  |  |  |  |  |  |  |
|  | Controllers | AC921 | M1 | M6 |  | M6 |  |  | M4 | M6 |  | M4 | M6 |  | M4 | M6 | 2x10^5^ | <50 | 518 (63.9) | 464-1,012 | - | - |
|  |  | AF942 | M3 | M3 |  | M3 | M3 |  | M3 | M5 |  | M3 | M5 |  | M3 | M5 | 9.9x10^5^ | <50 | 176 (89.6) | 51-1,048 | - | - |
|  |  | AG347 | M2 | M2 |  | M2 | M2 |  | M2 | M2 |  | M2 | M2 |  | M2 | M2 | 2.9x10^6^ | <50 | 518 (69.7) | 315-665 | - | - |
|  |  | AG934 | M3 | M3 |  | M3 | M3 |  | M3 | M5 |  | M3 | M5 |  | M3 | M5 | 1x10^5^ | <50 | 320 (66.6) | 330-567 | - | - |
|  |  |  |  |  |  |  |  |  |  |  |  |  |  |  |  |  |  |  |  |  |  |  |
| Vaccinated | Viremics | AF134 | M3 | M5 |  | M3 | M5 |  | M3 | M5 |  | M3 | M5 |  | M3 | M5 | 8.5x10^4^ | 2.3 - 69 × 10^3d)^ | 55 (95.5) | 20 - 40 | 100 | <100 |
|  |  | AF924 | M1 | M2 |  | M1 | M2 |  | M1 | M2 |  | M1 | M2 |  | M1 | M2 | 6.9x10^6^ | 0.41 - 2.5 × 10^4^ | 42 (97.6) | 111 - 231 | 800 | <100-200 |
|  |  | M770F | M1 | M3 |  | M1 | M3 |  | M1 | M3 |  | M1 | M3 |  | M1 | M3 | 2.1x10^5^ | <50 - 3.6 × 10^2e^ | 1,586 (8.3) | 1,300 – 2,356 | 800 | <100-200 |
|  |  | BD765B | M2 | M6 |  | M2 |  |  | M1 | M4 |  | M1 | M4 |  | M1 | M4 | 9.4x10^4^ | 0.0008 -1.6 × 10^5^ | 20 (97.6) | 86 | 200 | 200-800 |
|  |  | AG269 | M1 | M2 |  | M1 | M4 |  | M1 | M1 |  | M1 | M1 |  | M1 | M1 | 11.3x10^6^ | Dead^d)^ | 217 (83.4) | dead | 800 | dead |
|  | Controllers |  |  |  |  |  |  |  |  |  |  |  |  |  |  |  |  |  |  |  |  |  |
|  |  | AH484 | M3 | M4 |  | M3 |  |  | M1 | M3 |  | M1 | M3 |  | M1 | M3 | 2x10^4^ | <50 - 1.3 × 10^2^ | 561 (49.2) | 876-1,420 | 400 | 100-400 |
|  |  | AH776 | M3 | M3 |  | M3 |  |  | M1 | M3 |  | M1 | M3 |  | M1 | M3 | <50 | <50 | 1,156 (+44.1) | 487 - 2,399 | 800 | 100-800 |
|  |  | AG291 | M2 | M4 |  | M2 |  |  | M2 | M6 |  | M2 | M6 |  | M2 | M6 | 7.1x10^3^ | <50 | 1,476 (+0.4) | 1,130 - 2,270 | 1,600 | 200-400 |
|  |  | O854G | M1 | M3 |  | M1 | M3 |  | M1 | M3 |  | M1 | M3 |  | M1 | M3 | 1.1x10^4^ | <50 – 1.8 x 10^2^ | 594 (97.6) | 880 - 2,560 | 3,200 | 200-800 |

**TABLE S3**. **Summary of data reporting MHC haplotypes, viral load, CD4+ T cell counts and anti-Tat IgG antibodies in samples of control and vaccinated macaques.**

^a)^Viral load during the acute phase of the infection (2-4 weeks after challenge).

^b)^Viral load during the chronic phase of the infection (22-74 weeks after challenge).

^c)^CD4+ T cell counts as determined at week 4 after the challenge. The number in parenthesis indicates the percentage of decrease or increase (+) with respect the values of CD4+ T cell counts recorded on the day of challenge.

^d)^The vaccinated monkeys AG269and AF134 died at weeks 26 and 46 after the challenge, respectively. Similarly, the control macaques AC032 and AC739 died at weeks 40 and 46 after the challenge, respectively.
