# Supplementary material for: Biocompatible Anionic Polymeric Microspheres as Priming Delivery System for Effetive HIV/AIDS Tat-Based Vaccines
Source: PLoS One. 2014 Oct 30;9(10):e111360. doi: 10.1371/journal.pone.0111360 (PMC4214729; doi:10.1371/journal.pone.0111360)
Supplement: Table S4 — Impact of the MHC haplotype on acute viremia and disease progression in control and vaccinated macaques during the acute (2–4 weeks), post acute (8–16 weeks) and chronic (22–74) phase of the infection with SHIV89.6P. (DOCX) [file pone.0111360.s006.docx]

**TABLE S4. Impact of MHC IB haplotype on plasma viral load of control and vaccinated macaques during the acute (2-4 weeks), post acute (8-16 weeks) and chronic (22-74 weeks) phase of the infection following the challenge with SHIV89.6P.**

|  |  | Control monkeys | | | | |  | Vaccinated monkeys | | | | |
| --- | --- | --- | --- | --- | --- | --- | --- | --- | --- | --- | --- | --- |
| **MHC IB** | Phase of infection | Number of monkeys | Viral load (log_10_ copies/ml) | | | |  | Number of monkeys | Viral load (log_10_ copies/ml) | | | |
|  |  |  | Median | Std Error | Min | Max |  |  | Median | Std Error | Min | Max |
| H1 ^a)^ | Acute | 2 | 6.84 | 0.20 | 6.63 | 7.04 |  | 4 ^b)^ | 5.72 | 0.60 | 4.04 | 6.84 |
|  | Chronic |  | 3.63 | 0.42 | 3.21 | 4.04 |  |  | 1.39 | 0.75 | 0.85 | 4.02 |
| Non-H1 | Acute | 7 | 6.00 | 0.21 | 5.00 | 6.52 |  | 5 | 4.30 | 0.76 | 0.85 | 4.97 |
|  | Chronic |  | 1.38 | 0.43 | 0.85 | 3.47 |  |  | 1.10 | 0.67 | 0.85 | 3.93 |
| H2 | Acute | 2 | 6.75 | 0.29 | 6.46 | 7.04 |  | 3 | 4.97 | 0.87 | 3.85 | 6.84 |
|  | Chronic |  | 2.71 | 1.33 | 1.38 | 4.04 |  |  | 3.93 | 0.96 | 1.10 | 4.02 |
| Non-H2 | Acute | 7 | 6.00 | 0.23 | 5.00 | 6.63 |  | 6 | 4.62 | 0.75 | 0.85 | 6.11 |
|  | Chronic |  | 1.94 | 0.46 | 0.85 | 3.47 |  |  | 0.93 | 0.43 | 0.85 | 3.46 |
| H3 | Acute | 5 | 6.00 | 0.27 | 5.00 | 6.63 |  | 5 | 4.30 | 0.79 | 0.85 | 5.32 |
|  | Chronic |  | 1.94 | 0.53 | 0.85 | 3.37 |  |  | 1.01 | 0.50 | 0.85 | 3.46 |
| Non-H3 | Acute | 4 | 6.49 | 0.37 | 5.30 | 7.04 |  | 4 | 5.54 | 0.66 | 3.85 | 6.84 |
|  | Chronic |  | 2.43 | 0.78 | 0.85 | 4.04 |  |  | 2.51 | 0.87 | 0.85 | 4.02 |
| H4 | Acute | 1 | 6.52 |  | 6.52 | 6.52 |  | 1 | 6.11 |  | 6.11 | 6.11 |
|  | Chronic |  | 3.47 |  | 3.47 | 3.47 |  |  | 0.85 |  | 0.85 | 0.85 |
| Non-H4 | Acute | 8 | 6.07 | 0.24 | 5.00 | 7.04 |  | 8 | 4.62 | 0.60 | 0.85 | 6.84 |
|  | Chronic |  | 1.66 | 0.45 | 0.85 | 4.04 |  |  | 1.51 | 0.50 | 0.85 | 4.02 |
| H5 | Acute | 1 | 5.94 |  | 5.94 | 5.94 |  | 1 | 4.93 |  | 4.93 | 4.93 |
|  | Chronic |  | 3.37 |  | 3.37 | 3.37 |  |  | 3.46 |  | 3.46 | 3.46 |
| Non-H5 | Acute | 8 | 6.30 | 0.24 | 5.00 | 7.04 |  | 8 | 4.64 | 0.64 | 0.85 | 6.84 |
|  | Chronic |  | 1.66 | 0.46 | 0.85 | 4.04 |  |  | 1.06 | 0.49 | 0.85 | 4.02 |
| H6 | Acute | 2 | 5.72 | 0.42 | 5.30 | 6.15 |  | 0 |  |  |  |  |
|  | Chronic |  | 1.40 | 0.55 | 0.85 | 1.94 |  |  |  |  |  |  |
| Non-H6 | Acute | 7 | 6.46 | 0.25 | 5.00 | 7.04 |  | 9 | 4.93 | 0.57 | 0.85 | 6.84 |
|  | Chronic |  | 3.21 | 0.51 | 0.85 | 4.04 |  |  | 1.10 | 0.47 | 0.85 | 4.02 |
| Recombinant ^b)^ | Acute | 2 | 5.91 | 0.61 | 5.30 | 6.52 |  | 4 | 4.08 | 0.91 | 0.85 | 4.97 |
|  | Chronic |  | 2.16 | 1.31 | 0.85 | 3.47 |  |  | 1.06 | 0.74 | 0.85 | 3.93 |
| Non-recombinant | Acute | 7 | 6.15 | 0.24 | 5.00 | 7.04 |  | 5 | 5.32 | 0.48 | 4.04 | 6.84 |
|  | Chronic |  | 1.94 | 0.48 | 0.85 | 4.04 |  |  | 1.92 | 0.66 | 0.85 | 4.02 |

1. A significant association of the MHC IB M1 haplotype with viremia level was observed in the acute phase of the infection in two control macaques (Kruskal-Wallis Test, p= 0,0404), but not in 4 vaccinees (Kruskal-Wallis Test p= 0,7697^b)^).
