# Supplementary material for: Biocompatible Anionic Polymeric Microspheres as Priming Delivery System for Effetive HIV/AIDS Tat-Based Vaccines
Source: PLoS One. 2014 Oct 30;9(10):e111360. doi: 10.1371/journal.pone.0111360 (PMC4214729; doi:10.1371/journal.pone.0111360)
Supplement: Table S5 — Impact of MHC IB haplotype on antibody responses in vaccinated monkeys as observed during the acute (2–4 weeks), post acute (8–16 weeks) and chronic (22–74) phase of the infection with SHIV89.6p. (DOCX) [file pone.0111360.s007.docx]

**TABLE S5.** **Impact of MHC Class IB haplotype on the anti-Tat antibody responses detected in vaccinees during the acute (2-4 weeks), post acute (8-16 weeks), or chronic (22-74 weeks) phase of the infection following the challenge with SHIV89.6P.**

| MHC IB | Number of monkeys | Phases of Infection | Anti-Tat IgG titers | | |
| --- | --- | --- | --- | --- | --- |
|  | N |  | Median | Min | Max |
| H1 | 4 | Acute | 600 | 200 | 1,600 |
|  |  | Post-acute | 413 | 150 | 1,200 |
|  |  | Chronic | 206 | 81 | 400 |
| Non-H1 | 5 | Acute | 400 | 100 | 1,600 |
|  |  | Post-acute | 200 | 50 | 600 |
|  |  | Chronic | 225 | 50 | 575 |
| H2 | 3 | Acute | 200 | 100 | 400 |
|  |  | Post-acute | 425 | 50 | 600 |
|  |  | Chronic | 81 | 50 | 575 |
| Non-H2 | 6 | Acute | 600 | 200 | 1,600 |
|  |  | Post-acute | 213 | 150 | 1,200 |
|  |  | Chronic | 256 | 50 | 400 |
| H3 | 5 | Acute | 400 | 200 | 1,600 |
|  |  | Post-acute | 200 | 150 | 1,200 |
|  |  | Chronic | 225 | 50 | 313 |
| Non-H3 | 4 | Acute | 300 | 100 | 800 |
|  |  | Post-acute | 413 | 50 | 600 |
|  |  | Chronic | 241 | 50 | 575 |
| H4 | 1 | Acute | 800 | 800 | 800 |
|  |  | Post-acute | 400 | 400 | 400 |
|  |  | Chronic | 400 | 400 | 400 |
| Non-H4 | 8 | Acute | 400 | 100 | 1,600 |
|  |  | Post-acute | 213 | 50 | 1,200 |
|  |  | Chronic | 175 | 50 | 575 |
| H5 | 1 | Acute | 1,600 | 1,600 | 1,600 |
|  |  | Post-acute | 225 | 225 | 225 |
|  |  | Chronic | 50 | 50 | 50 |
| Non-H5 | 8 | Acute | 400 | 100 | 1,600 |
|  |  | Post-acute | 300 | 50 | 1,200 |
|  |  | Chronic | 256 | 50 | 575 |
| H6 | 0 |  |  |  |  |
| Non-H6 | 9 | Acute | 400 | 100 | 1,600 |
|  |  | Post-acute | 225 | 50 | 1,200 |
|  |  | Chronic | 225 | 50 | 575 |
| Recombinant | 4 | Acute | 300 | 100 | 400 |
|  |  | Post-acute | 175 | 50 | 600 |
|  |  | Chronic | 269 | 50 | 575 |
| Non-recombinant | 5 | Acute | 800 | 200 | 1,600 |
|  |  | Post-acute | 400 | 150 | 1,200 |
|  |  | Chronic | 125 | 50 | 400 |
